# To cut or not to cut? A prospective randomized controlled trial on short-term outcomes of the uncut Roux-en-Y reconstruction for gastric cancer

**DOI:** 10.1007/s00464-023-10067-0

**Published:** 2023-05-09

**Authors:** Hao Xu, Li Yang, Dian-Cai Zhang, Zheng Li, Qing-Ya Li, Lin-Jun Wang, Feng-Yuan Li, Wei-Zhi Wang, Yi-Wen Xia, Ze-Kuan Xu

**Affiliations:** 1 Division of Gastric Surgery, Department of General Surgery, The First Affiliated Hospital of Nanjing Medical University, Nanjing, Jiangsu China; 2 Jiangsu Key Lab of Cancer Biomarkers, Prevention and Treatment, Collaborative Innovation Center for Cancer Personalized Medicine, Nanjing Medical University, Nanjing, Jiangsu China

**Keywords:** Uncut Roux-en-Y reconstruction, Quality of life, Recanalization, Laparoscopic distal gastrectomy, Early gastric cancer

## Abstract

**Background:**

Roux-en-Y (R-Y) anastomoses have been widely used in distal gastrectomy, while the incidence of Roux stasis syndrome remains common. Uncut R-Y anastomosis maintains the neuromuscular continuity, thus avoiding the ectopic pacemaker of the Roux limb and reducing the occurrence of Roux stasis. However, retrospective studies of Uncut R-Y anastomosis remain scarce and randomized controlled trials have not been reported.

**Methods:**

We conducted a randomized controlled trial to compare the surgical safety, nutritional status, and postoperative quality of life (QOL) between uncut and classic Roux-en-Y (R-Y) reconstruction patients. Patients with Stage I gastric cancer were randomly enrolled and underwent laparoscopic distal gastrectomy followed by uncut or classic R-Y reconstruction. Body mass index and blood test were used to evaluate the nutritional status. QOL was evaluated using European Organization for Research and Treatment of Cancer QOL Questionnaire (STO22) and laboratory examinations at postoperative month (POM) 3, 6, 9, and 12. Computed tomography scanning was used to evaluate the skeletal muscle index (SMI) at POM 6 and 12. Endoscopy was performed at POM 12.

**Results:**

Operation time, blood loss, time to recovery, complication morbidities, and overall survival were similar between the two groups. Compared with the classic R-Y group, the uncut R-Y group displayed a significantly decreased QOL at POM 9, possibly due to loop recanalization, determined to be occupied 34.2% of the uncut R-Y group. Post-exclusion of recanalization, the QOL was still higher in the classic R-Y group than in the uncut R-Y group, despite their hemoglobin and total protein levels being better than those in the classic R-Y group. Preoperative pre-albumin level and impaired fasting glycemia significantly correlated with the postoperative recanalization.

**Conclusion:**

We found no significant benefit of uncut over classic R-Y reconstruction which challenges the superiority of the uncut R-Y reconstruction.

**Trial registration:**

ClinicalTrials.gov Identifier: NCT02644148.

**Supplementary Information:**

The online version contains supplementary material available at 10.1007/s00464-023-10067-0.

Gastric cancer (GC) is common worldwide, with > 1,000,000 cases and 783,000 deaths in 2018, ranking fifth among diagnosed cancers and third among cancer-mediated deaths [1]. Laparoscopic distal gastrectomy has been a standard and safe approach for both early [2–4] and advanced [5–9] distal GC. Billroth I, Billroth II, and Roux-en-Y (R-Y) anastomoses have been used in distal gastrectomy for > 130 years; however, there is no international consensus on digestive tract reconstruction after laparoscopic distal gastrectomy. Although R-Y is preferred for efficiently preventing bile reflux and its more favorable outcomes worldwide [10], the incidence of Roux stasis syndrome that manifests as nausea, vomiting, or abdominal distension is > 30% [11], as it decreases the patients’ quality of life (QOL).

In 1988, van Stiegman and Goff created a new method called “uncut Roux-en-Y reconstruction,” using linear staples to occlude the afferent jejunal lumen without cutting it [12]. Uncut R-Y anastomosis maintains the neuromuscular continuity, thus avoiding the ectopic pacemaker of the Roux limb after R-Y anastomosis and reducing the occurrence of Roux stasis [13]. Kim et al. claimed that this type of anastomosis constituted a favorable reconstructive laparoscopic procedure, since the uncut R-Y preserves the jejunum continuity and ensures neuronal transmission in electromyography while avoiding the emergence ectopic jejunal pacemakers [14].

However, retrospective studies of Uncut R-Y anastomosis remain scarce and randomized controlled trials have not been reported. Here, we conducted a prospective clinical randomized controlled study to document the short-term clinical outcomes and the 1-year QOL in GC patients who underwent laparoscopic distal gastrectomy with Roux-en-Y or uncut Roux-en-Y anastomosis. Health-related QOL is evaluated by the European Organization for Research and Treatment of Cancer (EORTC) QOL Questionnaire QLQ-STO22 [15, 16]. This study was registered in the NIH as NCT02644148.

## Methods

### Study design

This prospective, open-label, phase II, randomized controlled study was conducted between April 2016 and October 2019, at the First Affiliated Hospital of Nanjing Medical University. This study is to investigate the differences in short-term outcomes between classic R-Y and uncut R-Y anastomosis in laparoscopic gastrectomy for GC. This study followed the Consolidated Standards of Reporting Trials (CONSORT) reporting guideline and the protocol was approved by the institutional review and ethics committee of the First Affiliated Hospital of Nanjing Medical University (2015-SR-081). Informed consent was obtained from all patients prior to their enrollment in the study. The approved study protocol was available in Supplementary file 1.

### Participants

The inclusion criteria for enrollment were as follows: (1) aged 18–70 years; (2) bearing a distal gastric adenocarcinoma of > 5 cm from the cardia confirmed by endoscopic biopsy and suitable for distal gastrectomy; (3) with a clinical stage I tumor; (4) devoid of any mental illness; (5) able to fill out the EORTC questionnaires; (6) having provided written consent; and (7) available for follow-up until the end of the study.

The following patients were excluded from the study: (1) necessitating chemotherapy pre- or post-surgery according to the NCCN guidelines to avoid interference with the detection of postoperative QOL differences; (2) pregnant or breastfeeding women; (3) bearing ongoing infections; (4) bearing severe mental disorders; (5) bearing serious cardiovascular diseases, liver or kidney dysfunction (glutamic-pyruvic or glutamic-oxaloacetic transaminases and serum creatinine of > 300% and > 150% higher than normal, respectively), abnormal blood clotting function (mean prothrombin and activated partial thromboplastin higher than the normal limit of 50%), and neuropsychiatric disorders; (6) bearing other malignant tumors; (7) having requested to be excluded from the study; and (8) having had total gastrectomy due to unsuitable for distal gastrectomy.

### Randomization and data monitoring

To exclude the influence of different surgeons on the results, an interactive web-based response system deploying a central, dynamic, and stratified randomization procedure was used to assign eligible patients. The stratification factors of the randomization process were surgeons at our center. The randomization was conducted at the time of preparation for anastomosis after the completion of lymph node dissection. The enrolled patients were divided into two groups, an uncut R-Y and a classic R-Y group, without prior knowledge. The information of patients enrolled in this study was available in Supplementary file 2. Our research center (The First Affiliated Hospital of Nanjing Medical University, Nanjing, China) was responsible for this study.

### Eligibility of surgeons

Surgeons who met the following criteria qualified for this surgery: (1) had performed at least 500 distal gastrectomies with D2 lymphadenectomy by open or laparoscopic approaches, (2) had performed at least 300 gastrectomies for patients with GC annually at our center, and (3) had been trained strictly on GCP. All unedited video files of the surgical procedures involved in this trial were stored.

### Surgical technique

Surgery was conducted as previously reported [17]. Under general anesthesia, the patient was placed in the supine position. The surgeon stood on the patient’s left, the first assistant on their right, and the camera operator between the patient’s legs. After establishing pneumoperitoneum, five ports were generated, and an electro-laparoscope was introduced through the umbilical port. A D2 lymph node dissection as defined in the Japanese Gastric Cancer Treatment Guidelines was performed in this clinical trial [18].

#### Uncut R-Y method

After lymphadenectomy, specimens were removed from the abdominal cavity and confirmed with negative margins. Reconstruction was then initiated. The transverse colon was lifted to expose the ligament of Treitz, the jejunum at 20 cm distally to the ligament was marked with a thread and taken out, and an extracorporeal side-to-side anastomosis was generated. A stapler without a blade (Ethicon Endo-Surgery AKT45) was used to block the afferent jejunum. The small bowel was returned to the abdominal cavity and the pneumoperitoneum was reconstructed after closing the abdominal incision. The jejunum, 5 cm distally to the occlusive line, was anastomosed to the side of the residual stomach using a 60-mm linear stapler (Ethicon Endo-Surgery Echelon 60). Their common entry was then closed using another 60-mm linear stapler. A drainage tube was inserted after peritoneal irrigation.

#### Classic R-Y method

The difference between this and the previous procedure was that a linear stapler was used to block the afferent limbs. In the R-Y group, a normal linear stapler with a blade (Ethicon Endo-Surgery Echelon 60) was used.

The video illustrating Uncut R-Y and Classic R-Y anastomosis method was available in Supplementary files 3 and 4.

### Outcome measurements

**Fig. 1:**
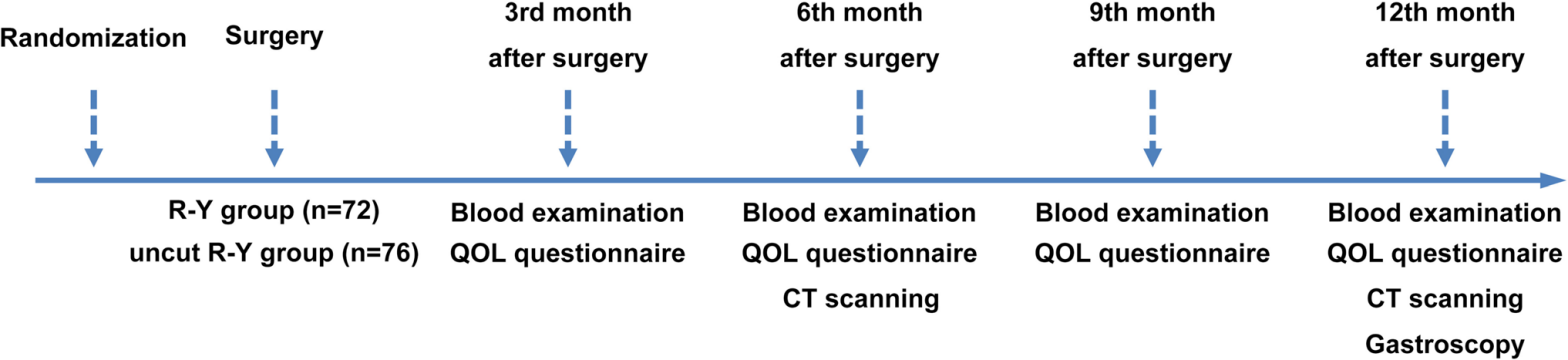
The patients’ follow-up schedules in R-Y group and uncut R-Y group

The clinical research coordinator at the data center communicated with the patient and help them fill EORTC QLQ-STO22 questionnaires. The surgeon reported the operation time, blood loss, lymph node retrieval, and pathological findings. The nutritional status of the patients was determined by routine blood and blood chemistry tests at POM 3, 6, 9, and 12. Endoscopic examinations were conducted at POM 6 and 12 according to the NCCN guidelines. For recanalization determination, endoscopy and gastrointestinal radiography results at POM 12 were used as guiding evidence. The patients’ follow-up schedules are shown in Fig. 1.

### Computed tomography (CT)-based analysis of skeletal muscle index (SMI) and visceral fat contents

In our study, CT-based analyses were performed using the OsiriX9 software by an experienced radiologist, blinded to the study. This researcher learned to identify the appropriate spinal level (Fig. 6). The skeletal muscle covering area (in cm^2^) was measured at the level of the fourth lumbar vertebra (L4). The surface area (in cm^2^) of the two consecutive images was used to calculate the average surface area. Areas with attenuation thresholds of − 29 to + 150 Hounsfield units (HU) were considered skeletal areas. $\left[ {{\text{SMI}} = \left( {\frac{skeletal\, area}{{height}}} \right)^{2} {\text{cm}}^{2} \;{\text{m}}^{ - 2} } \right]$. For visceral fat content calculation, tissues from − 50 to − 150 HU were identified as visceral fat at L4.

### Statistical analysis

The primary endpoint was the QOL score measured by QLQ-STO22 up to 12 months after surgery. According to our previous retrospective data from September 2014 to August 2018 (*n* = 228) [17], the means of QOL scores measured by QLQ-STO22 in uncut Roux-en-Y anastomosis and classic Roux-en-Y anastomosis were 23 and 26 with group standard deviations of 5.5 and 7.5, respectively. Sample size was calculated using PASS 11. A sample size of 76 per group was calculated as necessary for 80% power to detect a difference between the two groups with a significance level (alpha) of 0.05 using a two-sided two-sample *t* test. Assuming a dropout rate of 10%, sample size was increased to 85 patients for each group.

Chi-squared analysis was used to compare categorical data, and Student’s *t* test was used for continuous variables. Linear mixed models were used to compare the longitudinal scores of the QLQ-STO22. Data are presented as the mean ± standard deviation for continuous variables. All *p *values were two-sided, and *p *values less than 0.05 indicated statistically significant differences. All statistical analyses were performed using SPSS ver. 22 for Windows (SPSS Inc., Chicago, IL, USA).

Participants who were randomly assigned but did not undergo surgery were excluded from all analyses (patients without signing informed consent were also excluded).

## Results

### Patient recruitment

**Fig. 2:**
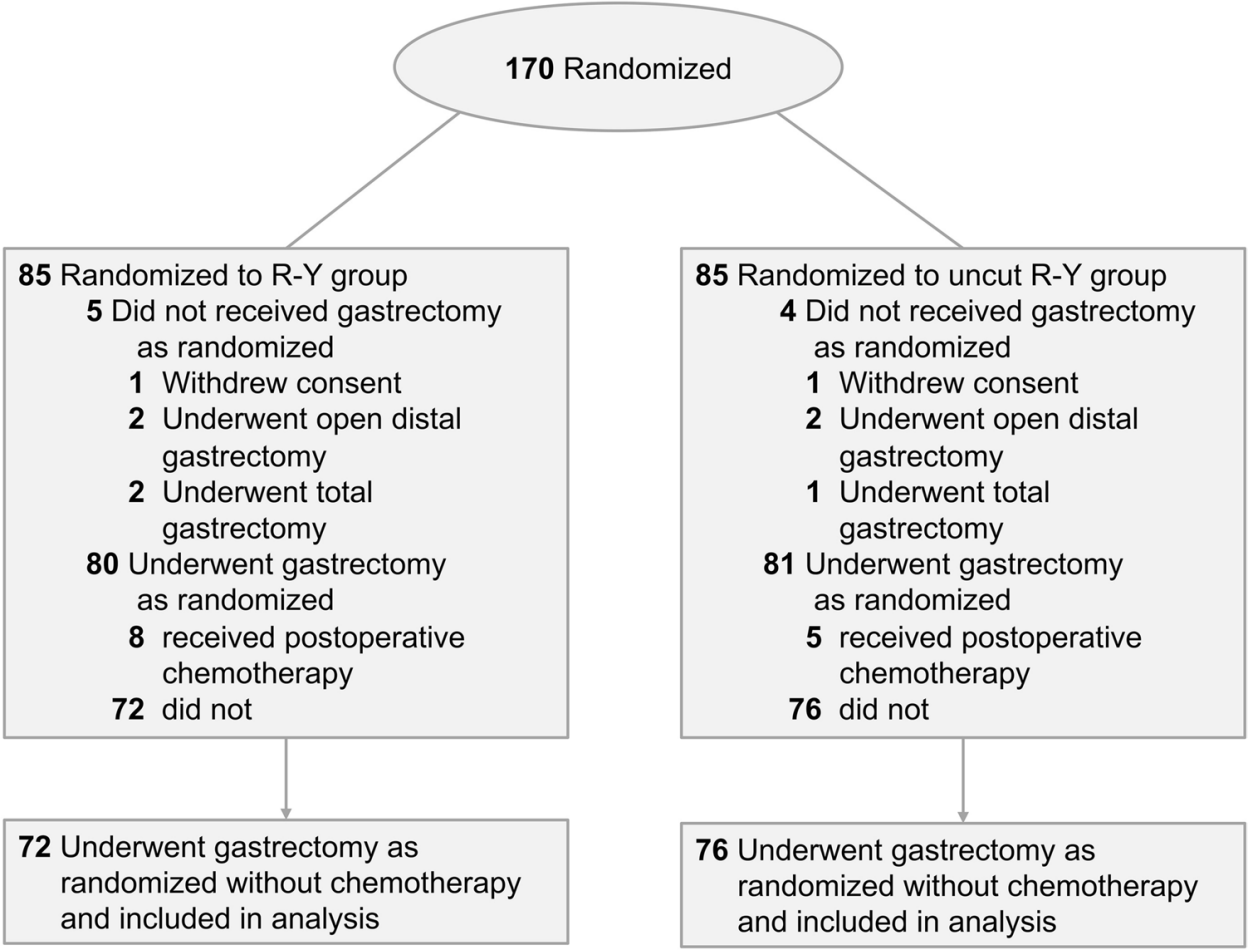
Flow diagram of R-Y group and uncut R-Y group in this study

Between April 2016 and October 2019, 170 patients were registered prospectively; 85 patients were randomly assigned to the R-Y group and 85 patients to the uncut R-Y group. As shown in Fig. 2, patients who did not sign the informed consent form, underwent total gastrectomy, open surgery, or received postoperative chemotherapy were excluded. Overall, 76 patients in the uncut R-Y and 72 in the R-Y group were included for downstream analysis. As shown in Table 1, there was no significant difference in the baseline data of the two groups of patients, such as the American Society of Anesthesiologists score, sex distribution, and age. There was no significant difference in body mass index (BMI) either (22.90 ± 2.98 kg/m^2^ in the R-Y vs. 23.20 ± 2.70 kg/m^2^ in the uncut R-Y group; *p* = 0.543). This indicated that there was no statistical difference in the nutritional status of the two groups of patients, thus permitting a comparison of the nutritional status between the two anastomosis methods. Moreover, the patients’ clinical stage showed no significant difference between the two groups.

**Table 1:** Comparison of patient characteristics between classic Roux-en-Y (R-Y) group and Uncut Roux-en-Y (Uncut R-Y) group

	R-Y group	Uncut R-Y group	*p* value
	(*n* = 72)	(*n* = 76)	
Gender (M/F)	39/33	43/33	0.869
Age	56.17 ± 11.20	57.86 ± 9.99	0.334
Body mass index			
(kg/m^2^)	22.90 ± 2.98	23.20 ± 2.70	0.543
ASA score			
I	44	40	0.323
II	28	36	
c stage			0.402
T1N0M0	57	53	
T1N1M0	6	8	
T2N0M0	9	15	
pT stage			
T1	67	73	0.563
T2	2	2	
T3	3	1	
pN stage			
N0	65	70	0.847
N1	3	2	
N2	3	2	
N3	1	2	
p stage			
IA	56	61	0.893
IB	9	9	
IIA	3	3	
IIB	3	3	
IIIB	1	0	
Pathological differentiation			
Well moderate	32	29	0.205
Poor	40	47	

### Short-term outcome of surgery

Surgical and short-term recovery outcomes are presented in Table 2. The operating time (167.28 ± 37.80 min vs. 175.22 ± 38.21 min; *p* = 0.212), bleeding amount (41.37 ± 15.27 min vs. 49.48 ± 31.34 min; *p* = 0.104), number of lymph nodes retrieved (40.63 ± 11.16 vs. 37.58 ± 12.30; *p* = 0.107), and number of positively retrieved lymph nodes (0.36 ± 1.33 vs. 0.51 ± 1.60; *p* = 0.553) were similar in both groups. The time required to perform the anastomosis in the R-Y group was shorter than that in the uncut R-Y group (20.62 ± 7.55 min vs. 24.26 ± 8.50 min; *p* = 0.029). As for the short-term recovery course, the time to first flatus was shorter in the uncut R-Y group than in the R-Y group (69.29 ± 23.65 h vs. 61.93 ± 22.54 h; *p* = 0.049). There was no significant difference between the two groups regarding the times to ambulation (35.61 ± 19.63 h vs. 36.69 ± 15.03 h; *p* = 0.857), first liquid intake (3.76 ± 0.93 days vs. 3.64 ± 0.81 days; *p* = 0.303), first meal (4.85 ± 0.82 days vs. 4.69 ± 0.92 days, *p* = 0.164), drainage tube extraction (6.00 ± 0.90 days vs. 5.87 ± 1.24 days, *p* = 0.317), and postoperative hospital stay (7.85 ± 1.68 days vs. 7.87 ± 1.53 days, *p* = 0.937). The expenses between the two groups were similar (65,541.32 ± 8,489.66 CNY vs. 65,474.41 ± 7,624.62 CNY; *p* = 0.962).

**Table 2:** Comparison of the surgical and short-term recovery outcomes between classic Roux-en-Y (R-Y) group and Uncut Roux-en-Y (Uncut R-Y) group

	R-Y group (*n* = 72)	Uncut R-Y group (*n* = 76)	*p* value
Operating time (min)	167.27 ± 37.80	175.22 ± 38.21	0.212
Anastomotic time (min)	20.62 ± 7.55	24.26 ± 8.50	0.029*
Bleeding (mL)	41.37 ± 15.27	49.48 ± 31.34	0.104
No. of retrieval lymph nodes	40.63 ± 11.16	37.58 ± 12.30	0.107
Positive no. of retrieval lymph nodes	0.36 ± 1.33	0.51 ± 1.60	0.553
Time to ambulation (h)	35.61 ± 19.63	36.69 ± 15.03	0.857
Time to first flatus (h)	69.29 ± 23.65	61.93 ± 22.54	0.049*
Time to first liquid intake (day)	3.76 ± 0.93	3.64 ± 0.81	0.303
Time to first meal (day)	4.85 ± 0.82	4.69 ± 0.92	0.164
Time to abdominal drainage tube extraction (day)	6.00 ± 0.90	5.87 ± 1.24	0.317
Postoperative hospital stays (day)	7.85 ± 1.68	7.87 ± 1.53	0.937
Postoperative complications			0.489
Total	4 (5.56%)	7 (9.21%)	
I	1 (1.39%)	1 (1.32%)	
II	3 (4.17%)	4 (5.26%)	
III	0 (0.00%)	2 (2.63%)	
Hospitalization expenses (¥)	65,541.32 ± 8489.66	65,474.41 ± 7624.62	0.962

*p < 0.05

In terms of postoperative complications, the morbidity rates in the R-Y group were 5.6% (4/72) and 9.2% (7/76) in the uncut R-Y group (*p* = 0.489). However, the complications in the latter appeared more serious than those in the R-Y group, and 2 grade III cases were identified in the uncut R-Y group.

### Survival

As patients enrolled in our study were mainly early stages GC, survival was not significantly different between the uncut R-Y and classic R-Y groups. These results are shown in Supplementary Fig. 1.

### Quality of life

**Fig. 3:**
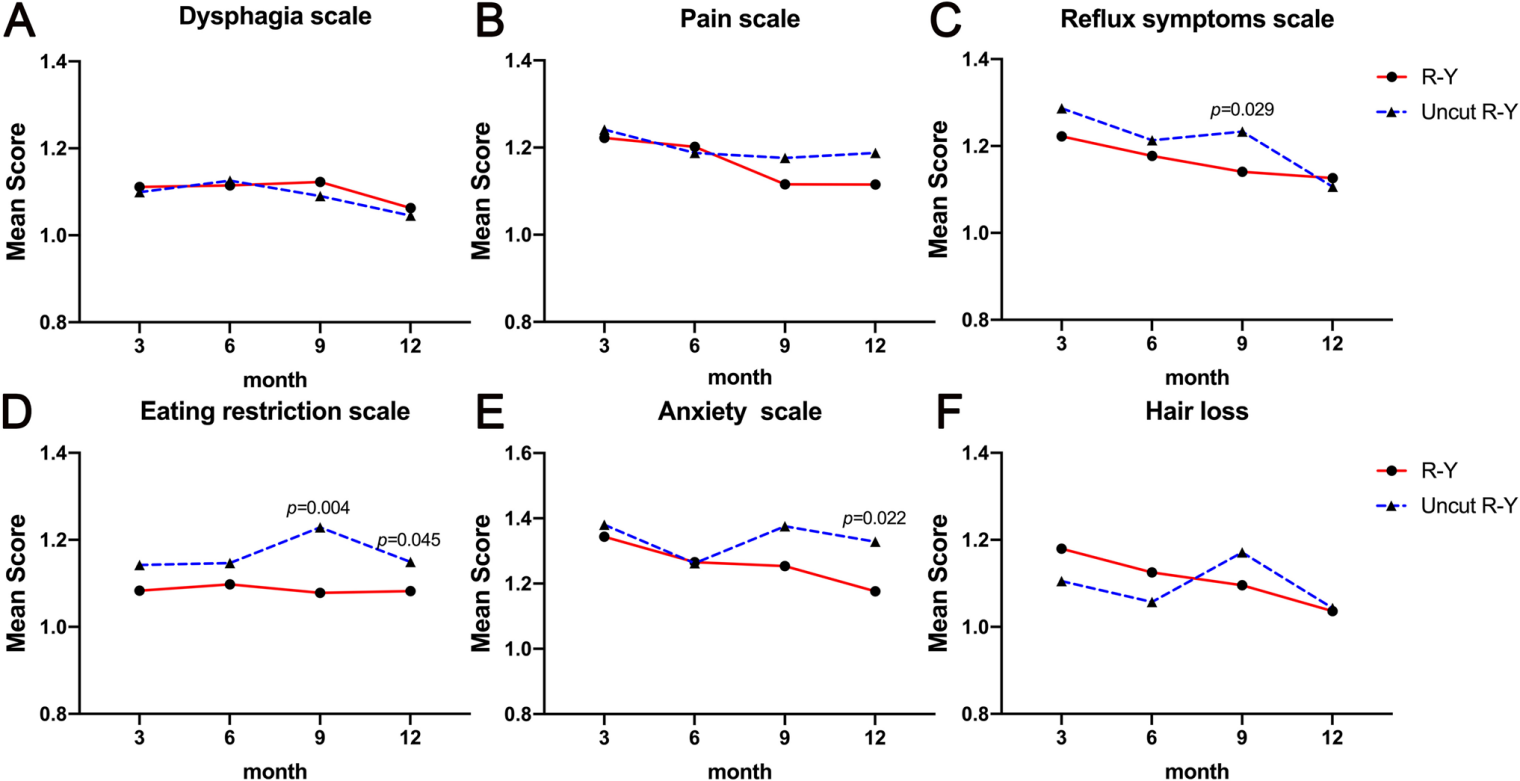
Quality of life scores according to QLQ-STO22 questionnaires of patients in R-Y group and uncut R-Y group on the 3rd, 6th, 9th, and 12th month after surgery, including dysphagia scale (**A**), pain scale (**B**), reflux symptoms scale (**C**), eating restriction scale (**D**), anxiety scale (**E**), and hair loss (**F**)

We scored the QLQ-STO22 questionnaires of the two groups of patients on the 3rd, 6th, 9th, and 12th month after surgery. The conditioned scores of the QLQ-STO22 are shown in Fig. 3. There was no significant difference in the dysphagia scale, pain scale, or hair loss between the R-Y and uncut R-Y groups. Regarding reflux symptoms, eating restrictions, and anxiety, patients in the uncut R-Y group were more likely to encounter QOL issues (detailed data are listed in Supplementary Table 1). Interestingly, these differences gradually appeared from the 9th-month post-surgery.

Studies have shown that limb recanalization should be considered while evaluating the performance of the uncut R-Y anastomosis method [17]. Animal experiments and studies by other scholars have shown that recanalization may occur after uncut R-Y anastomosis. However, the specific recanalization ratio and recanalization time lack high-quality clinical evidence. Current guidelines tend to review endoscopy one year after surgery and perform gastrointestinal angiography when necessary. Therefore, existing studies often find that patients undergoing uncut R-Y anastomosis exhibit recanalization after one year. Uncut R-Y is beneficial for avoiding the development of Roux stasis syndrome; therefore, the QOL of its patients may be better after surgery. We speculate that the decline in the QOL of Uncut R-Y group in the 9th month after surgery may have been caused by recanalization.

**Fig. 4:**
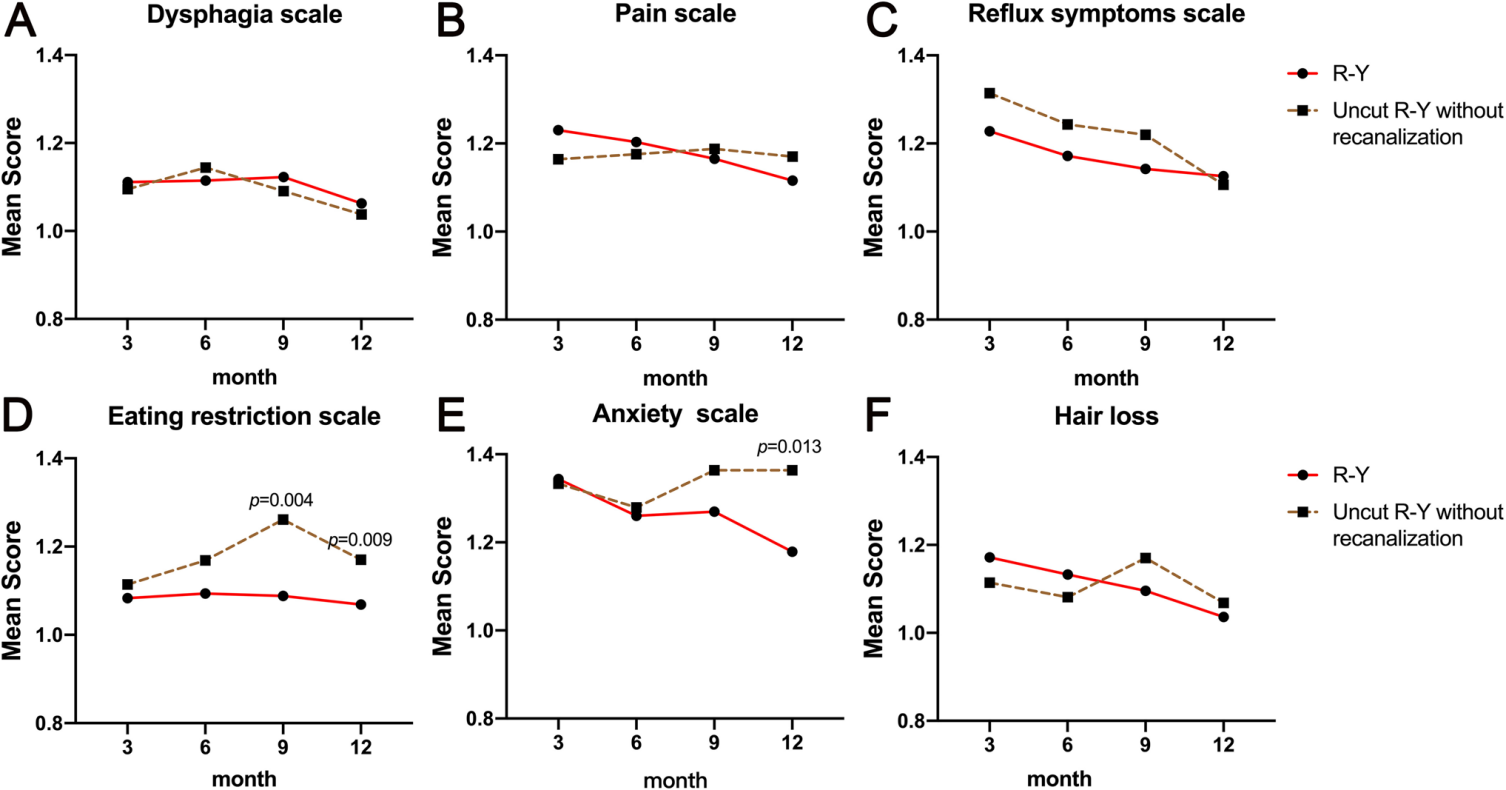
Quality of life scores according to QLQ-STO22 questionnaires of patients in R-Y group and uncut R-Y group after exclusion of those with recanalization on the 3rd, 6th, 9th, and 12th month after surgery, including dysphagia scale (**A**), pain scale (**B**), reflux symptoms scale (**C**), eating restriction scale (**D**), anxiety scale (**E**), and hair loss (**F**)

**Fig. 5:**
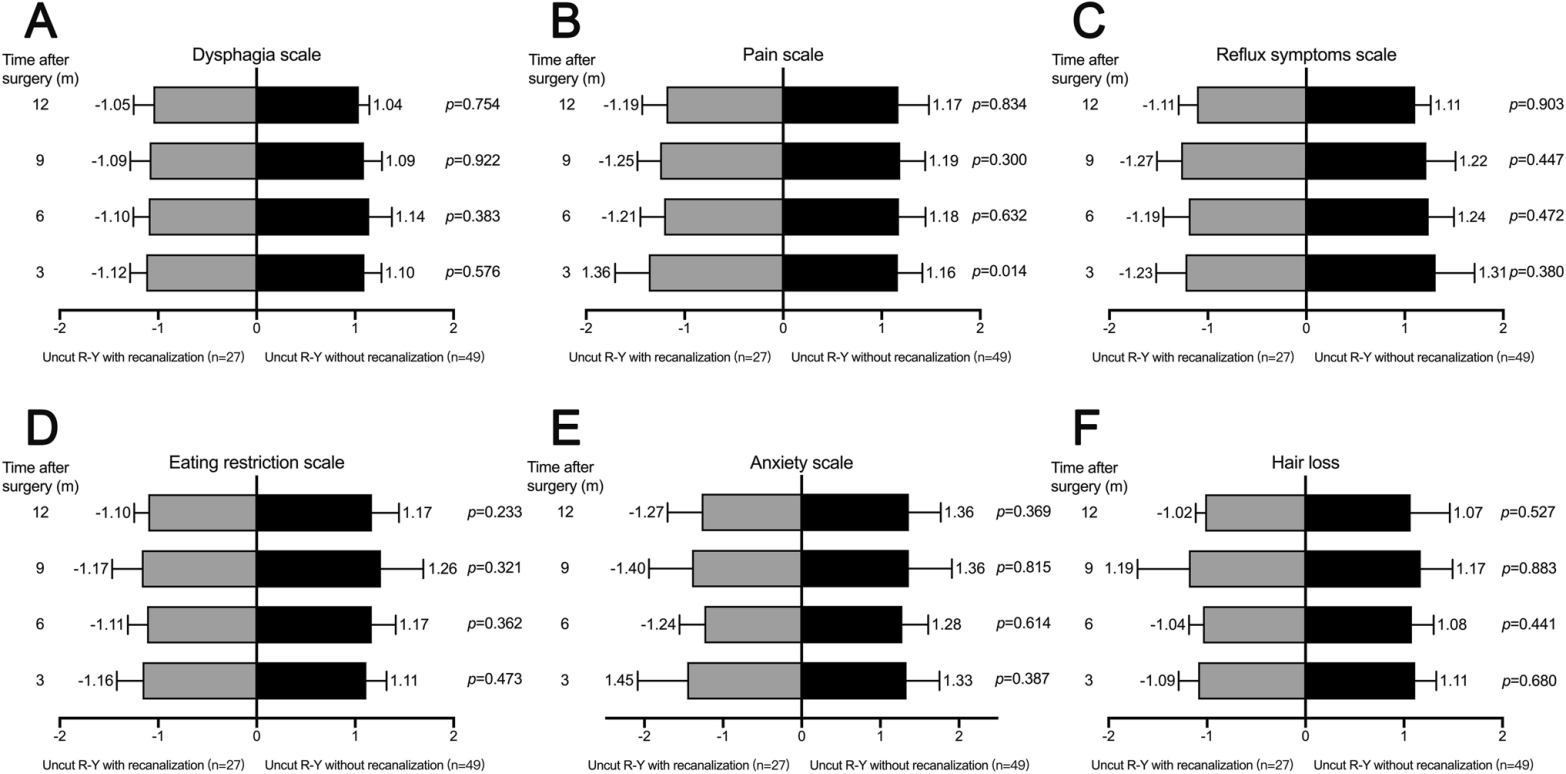
Quality of life scores according to QLQ-STO22 questionnaires of patients in uncut R-Y group with or without recanalization, including dysphagia scale (**A**), pain scale (**B**), reflux symptoms scale (**C**), eating restriction scale (**D**), anxiety scale (**E**), and hair loss (**F**)

All patients in the uncut R-Y group underwent gastroscopy or upper gastrointestinal radiography, which showed that 27 (35.5%) of the patients enrolled in the uncut R-Y group developed afferent limb recanalization. To analyze the impact of recanalization on the QOL, we re-analyzed the QOL of the two patient groups after exclusion of those with recanalization (Fig. 4). In this case, the differences of the QOL scores between the two groups of patients did not change significantly, and the QOL in the R-Y anastomosis group was still better. To further confirm the importance of limb recanalization, we analyzed the QOL scores of recanalized and non-recanalized patients in the uncut R-Y group. As shown in Fig. 5, recanalization did not affect the QOL scores in the uncut R-Y group, which indicated that, regardless of whether recanalization occurred, the QOL of patients in the uncut R-Y group was worse than that of patients in the R-Y group (detailed data are listed in Supplementary Table 2).

### Nutritional status

Results of routine blood tests, blood chemistry, and tumor marker changes within one year after the operation are listed in Supplementary Tables 3–7. We analyzed laboratory tests and BMI pre- and at POM 3, 6, 9, and 12. Results showed that magnesium (0.88 ± 0.07 mM vs. 0.94 ± 0.11 mM; *p* = 0.002) at POM 3, platelet [(200.75 ± 52.98) × 10^9^ L^−1^ vs. (180.56 ± 47.46) × 10^9^ L^−1^; *p* = 0.037] at POM 6, platelet [(199.82 ± 60.81) × 10^9^ L^−1^ vs. (175.12 ± 50.11) × 10^9^ L^−1^; *p* = 0.046], total protein (68.90 ± 5.25 g/L vs. 71.86 ± 4.53 g/L; *p* = 0.008) and globulin (26.68 ± 3.68 g/L vs. 28.67 ± 3.83 g/L; *p* = 0.022) at POM 9, and potassium (4.11 ± 0.55 mM vs. 4.29 ± 0.34 mM; *p* = 0.045) at POM 12 (R-Y vs. uncut R-Y, respectively) showed significant differences. However, these different data are still maintained at normal range.

As mentioned above, we found that the QOL of the two patient groups was significantly different at POM 9, and we speculate that this phenomenon is related to recanalization. The results of the laboratory examination also showed that the differences between the two groups of patients were more obvious at POM 9. Therefore, we excluded patients who had undergone recanalization and analyzed the results of the laboratory tests. As shown in Supplementary Tables 8–12, indirect bilirubin (7.91 ± 2.82 μM vs. 9.15 ± 3.76 μM; *p* = 0.040) before surgery, white blood cell [(5.25 ± 1.18) × 10^9^ L^−1^ vs. (5.91 ± 1.65) × 10^9^ L^−1^; *p* = 0.026] and magnesium (0.88 ± 0.07 mM vs. 0.93 ± 0.14 mM; *p* = 0.018) at POM 3, hemoglobin (130.03 ± 12.88 g/L vs. 136.97 ± 13.33 g/L; *p* = 0.042), red blood cell [(4.12 ± 0.44) × 10^12^ L^−1^ vs. (4.42 ± 0.51) × 10^12^ L^−1^; *p* = 0.014], total protein (68.90 ± 5.25 g/L vs. 72.03 ± 4.75 g/L; *p* = 0.017), and globulin (26.68 ± 3.68 g/L vs. 28.77 ± 4.08 g/L, *p* = 0.036) at POM 6 (R-Y vs. uncut R-Y, respectively) showed significant differences while remaining within the normal range.

**Fig. 6:**
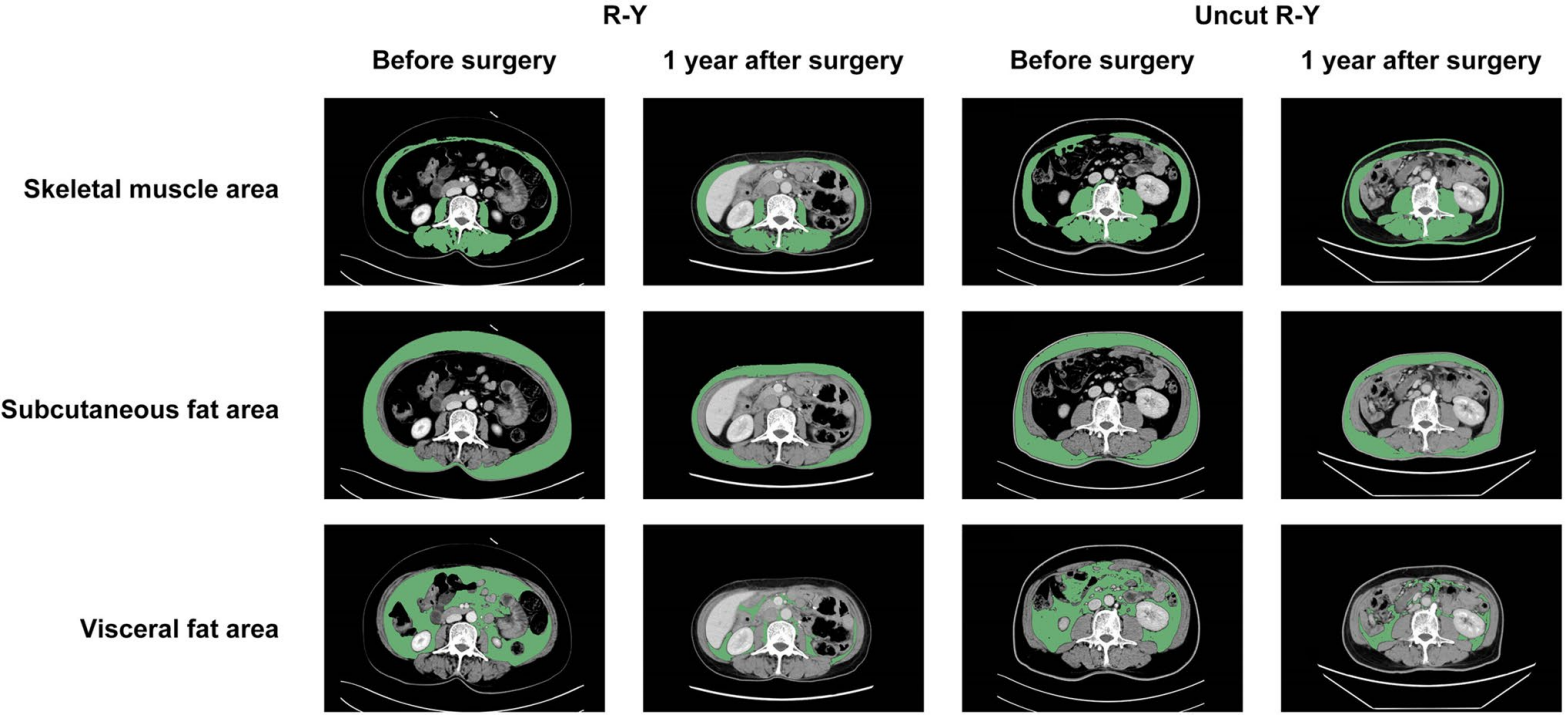
Representative image of skeletal muscle area, subcutaneous fat area, and visceral fat area from CT-based analyses at the level of the fourth lumbar vertebra (L4)

We also measured the CT-based SMI and visceral and subcutaneous fat contents of the patients to further uncover differences in nutritional status between the two groups. As shown in Fig. 6, with the help of software, we marked the calculation areas of skeletal muscle, visceral fat, and subcutaneous fat on the CT film at the L4 level. The results identified no significant difference between the R-Y and uncut R-Y groups in BMI, SMI, and visceral and subcutaneous fat one-year post-surgery (Table 3). The SMI of the uncut R-Y group was significantly smaller than that of the R-Y group before surgery (*p* = 0.040). In general, patients experienced variable degrees of SMI and decreased visceral and subcutaneous fat at one year after distal gastrectomy. This finding indicates that gastrectomy is the main cause of changes in the nutritional status of patients. The anastomosis approach did not influence these indicators significantly.

**Table 3:** Results of BMI, visceral fat area, subcutaneous fat area, visceral fat index, skeletal muscle area, and skeletal muscle index before surgery and at 1 year after surgery

		Group 1	Group 2	Group 3	Group 4	*p* value		
		R-Y group (n = 72)	Uncut R-Y group (n = 76)	Uncut R-Y with recanalization (n = 27)	Uncut R-Y without recanalization (n = 49)	1 vs 2	1 vs 4	3 vs 4
Before surgery	BMI (kg/m^2^)	22.90 ± 2.98	23.20 ± 2.7	23.07 ± 3.01	23.27 ± 2.56	0.543	0.495	0.761
	Visceral fat area (cm^2^)	100.62 ± 45.73	103.26 ± 48.94	105.17 ± 53.61	102.24 ± 46.9	0.753	0.861	0.823
	Subcutaneous fat area (cm^2^)	158.76 ± 67.32	150.64 ± 61.42	164.92 ± 77.48	142.97 ± 50.23	0.478	0.200	0.178
	Visceral fat index	0.68 ± 0.26	0.72 ± 0.31	0.69 ± 0.33	0.74 ± 0.29	0.371	0.252	0.530
	Skeletal muscle area (cm^2^)	119.68 ± 27.17	125.66 ± 29.81	117.10 ± 28.5	130.25 ± 29.81	0.237	0.063	0.095
	Skeletal muscle index (cm^2^/m^2^)	42.98 ± 8.05	46.42 ± 9.32	44.33 ± 9.1	47.46 ± 9.37	0.040*	0.015*	0.222
1 year after surgery	BMI (kg/m^2^)	20.92 ± 1.62	21.49 ± 2.55	21.65 ± 3.15	21.42 ± 2.34	0.368	0.417	0.816
	Visceral fat area (cm^2^)	53.92 ± 33.29	57.55 ± 39.15	51.00 ± 33.75	62.46 ± 42.61	0.611	0.314	0.283
	Subcutaneous fat area (cm^2^)	112.92 ± 47.95	113.39 ± 56.39	110.95 ± 59.88	115.23 ± 54.52	0.964	0.841	0.781
	Visceral fat index	0.49 ± 0.23	0.52 ± 0.25	0.49 ± 0.21	0.54 ± 0.27	0.598	0.406	0.446
	Skeletal muscle area (cm^2^)	117.70 ± 27.21	119.94 ± 28.12	111.91 ± 27.38	126.15 ± 27.51	0.907	0.180	0.062
	Skeletal muscle index (cm^2^/m^2^)	42.92 ± 7.71	44.63 ± 8.38	42.87 ± 8.41	45.88 ± 8.26	0.558	0.124	0.200

*p < 0.05

### Risk factor of recanalization in uncut R-Y group

Using gastroscopy or upper gastrointestinal radiography, we found that 35.5% (27 out of 76) of patients enrolled in the R-Y group developed afferent limb recanalization. Therefore, we further analyzed the risk factors underlying afferent limb recanalization. Using univariate and multivariate analyses, we found that preoperative pre-albumin level and impaired fasting glucose were significantly correlated with postoperative recanalization (Table 4), which indicated that uncut R-Y reconstruction for these patients needs to be performed with caution.

**Table 4:** Univariate analysis and multivariate analysis of the risk factor of afferent limb recanalization

	Uncut R-Y without recanalization (*n* = 49)	Uncut R-Y with recanalization (*n* = 27)	Univariate analysis *p* values	Multivariate analysis		
				Odds ratio	95% CI	*p* values
Age (years)			0.413			
< 60	24	17				
≥ 60	25	10				
ASA score			0.562			
I	27	13				
II	22	14				
BMI			0.945			
< 23	24	13				
≥ 23	25	14				
Operation time (min)			0.945			
< 165	25	14				
≥ 165	24	13				
3D laparoscopy			0.700			
Yes	16	10				
No	33	17				
Impaired fasting glucose			0.023*	6.666	1.397–31.811	0.017*
Yes	3	7				
No	46	20				
Preoperative SMI (cm^2^/m^2^)			0.233			
Higher half	22	16				
Lower half	27	11				
Preoperative Hemoglobin (g/L)			0.233			
Higher half	27	11				
Lower half	22	16				
Preoperative albumin (g/L)			0.473			
Higher half	26	12				
Lower half	23	15				
Preoperative Pre-albumin (g/L)			0.010*	4.310	1.464–12.658	0.008**
Higher half	30	8				
Lower half	19	19				

*p < 0.05, **p < 0.01

### Gastric emptying, residual gastritis, and reflux esophagitis assessment

To analyze the patients’ gastric emptying, residual gastritis, and reflux esophagitis, we collected the endoscopic examination results of the two groups of patients’ 1-year post-surgery. As shown in Table 5, 12.50% (9/72) of the patients in the R-Y group and 17.11% (13/76) of the patients in the uncut R-Y group appeared to have food leftovers under endoscopic observation. In addition, the incidence of residual gastritis in the R-Y group and the uncut R-Y group were 19.44% (14/72) and 21.05% (20/76), respectively, while the incidence of reflux esophagitis was 12.50% (9/72) and 13.16% (10/76). No statistically significant difference was found.

**Table 5:** Gastric emptying disorder, residual gastritis, and reflux esophagitis between R-Y group and Uncut R-Y group

	R-Y group (*n* = 72)	Uncut R-Y group (*n* = 76)	*p* value
Gastric emptying disorder	12.50% (9/72)	17.11% (13/76)	0.493
Residual gastritis	19.44% (14/72)	21.05% (20/76)	0.841
Reflux esophagitis	12.50% (9/72)	13.16% (10/76)	> 0.999

## Discussion

In this randomized controlled trial, we compared the surgical safety, nutritional status, and postoperative quality of life (QOL) between uncut and classic R-Y reconstruction patients. We confirmed that the operation time, blood loss, time to recovery, complication morbidities, and overall survival showed no significant difference. However, different from the original intention of uncut R-Y, we found that the QOL of the uncut R-Y group showed no better than that of the classic R-Y group. Moreover, the uncut R-Y group displayed a significantly decreased QOL at POM 9, possibly due to loop recanalization. Besides, preoperative pre-albumin level and impaired fasting glycemia significantly correlated with the postoperative recanalization. In brief, no significant benefit of uncut over classic R-Y reconstruction was found in our study, which challenges the superiority of the uncut R-Y reconstruction.

Previous studies of KLASS-01, CLASS-01, and JSES confirmed radical laparoscopic resection as safe and efficient for distal GC, with complication rates in KLASS-01 and JSES of 15.2% and 8.6%, respectively [2–4]. For advanced GC the rate was 15.2% (CLASS-01 trial) [9]. The overall rate in our study was 7.4%, with the R-Y group being at 5.6% and the uncut R-Y group at 9.2%. Sah et al. reported a complication morbidity of 20.8% in the uncut R-Y and 33.7% in the R-Y group; *p* = 0.028 [19]. The high complication rated reported by Sah et al. may be due to its retrospective design and the inclusion of both laparoscopic and open surgery. Other studies bore a morbidity of 9.8% in the uncut and 21.8% in the classic R-Y [14] or an overall morbidity of 4.84% [20]. Several studies have underscored the importance of analyzing complications and their incidence, which we believe was lower in our institution due to our surgical proficiency. We maintain a fixed surgical team and standardized surgical procedures, which, alongside screening patients’ physical condition before enrollment, can greatly reduce complications occurrence.

Studies by Li et al. [21] and Parket al. [14] evaluated serum hemoglobin, total protein, and albumin levels at the one-year follow-up post-surgery and found it similar in the two reconstruction procedures. Our results showed that only magnesium, platelet, total protein, globulin, and potassium levels before or at different time points post-surgery showed small, yet significant, differences, albeit with normal values. This indicates that laboratory examinations may not be sensitive enough to uncover the potential effects of R-Y and uncut R-Y on nutritional status.

The strength of this trial was the longitudinal comparison of the patients’ QOL. So far, comparisons between approaches were mostly performed at one time point. Yasuda et al. [22] and Ikenaga [23] compared QOL measurements at a single time point. Questionnaires used for the evaluation are considered to have characteristics of reliability, reproducibility, feasibility, and clinical validity. QLQ-STO22 has been translated in several different languages with relative validation studies in the corresponding countries and therefore was employed in our study. We found no significant difference of dysphagia scale, pain scale, or hair loss between R-Y and uncut R-Y groups. And the reflux symptoms scale, eating restriction scale, and anxiety scale showed a deteriorating trend from the 9th month after surgery. Our results seemed to be inconsistent with those reported in other literature, that is, the uncut R-Y anastomosis did not improve the quality of life than the R-Y anastomosis group, especially for the pain scale that related to Roux’s stasis. Moreover, from the 9th month, the pain level in the uncut R-Y group lagged behind the one in the R-Y group. As mentioned in the previous article, limb recanalization will occur in uncut R-Y anastomosis from 3 to 6 months after surgery. After limb recanalization occurs, the uncut R-Y anastomosis will be similar to the R-Y anastomosis, so that ectopic pacing points may appear on Roux limb to cause Roux stasis syndrome and other subjective feelings that may lead to a decline in the QOL. We believe that the decline in the QOL of patients in the uncut R-Y group is likely to be related to limb recanalization, since patients with uncut R-Y theoretically have a better QOL at the beginning.

For recanalization evaluation in our study, we used gastrointestinal angiography and endoscopy to determine the presence of limb recanalization. The flow of contrast medium in both directions after passing through the gastrointestinal anastomosis during the contrast examination indicated limb recanalization. Similarly, the unobstructed passage of the endoscope through the anastomosis toward the proximal bowel indicated limb recanalization.

The identification of recanalization after uncut R-Y has been reported previously. In contrast, Wang et al. [20] reported no recanalization after uncut R-Y reconstruction. Recanalization at the site of afferent closure after uncut R-Y reconstruction may increase the incidence of alkaline reflux gastritis and esophagitis and decrease the QOL. Chen et al. explained that recanalization was more common after 3 months, and Yang et al. concluded that the incidence rate of recanalization after uncut R-Y reconstruction reached 13.0% [17]. Wu et al. reported that all 20 experimental pigs presented recanalization of the jejunum closure one month after the operation and limb recanalization occurred in 50% (5/10) of patients’ 6-month post-surgery. The number of row lines in the staple seems to be unrelated to recanalization rates [24]. We uncovered a 35.5% recanalization incidence, i.e., substantially higher than that reported in other studies.

The following points should be considered. First, the distance from the gastrointestinal anastomosis to the occlusion may have affected the judgment of recanalization. When the distance between the occlusion and gastrointestinal anastomosis reaches 5 cm, it may cause the concentration of contrast agents and the long distance for the endoscope push forward, thus resembling and being mistaken for a limb recanalization. We controlled this distance to be 2-3 cm. Such an operation cannot reduce the recanalization rate, suggesting that the uncut R-Y has a certain recanalization possibility. Second, we used a non-bladed 2-row stapler, which may cause recanalization. Modified procedures have been used to reinforce the occlusion and reduce the recanalization rate. These include using non-bladed six-row linear staples, 4–5 seromuscular sutures annularly around the jejunal wall, tightly tied 3–0 polypropylene, and suturing of the serosal layers of the upper and lower jejunum at the occlusion site after jejunum ligature. In addition, a prospective randomized controlled study may eliminate deviations only to a certain extent. We further analyzed the risk factor of afferent limb recanalization and found that preoperative pre-albumin level and preoperative impaired fasting glucose significantly correlated with the postoperative recanalization, indicating that uncut R-Y reconstruction for these patients needs to be performed cautiously.

An inherent weakness was that this is a single-center trial. Nevertheless, R-Y reconstruction has not been a standard and universal treatment for GC. Therefore, a multi-institutional study is urgently needed. Another limitation was the insufficient sample size. We will continue to recruit more patients to solidify whether uncut R-Y reconstruction will improve QOL or bear long-term complications, such as reflux gastritis/esophagitis, delayed gastric emptying, Roux stasis syndrome, and reduced survival time. In addition, the combined use of the QLQC30 and QLQ-STO22 needs to be more thoroughly considered.

Taken together, we found no benefit differences between the two reconstruction methods, while the R-Y group bears advantages over the uncut R-Y group in terms of QOL and nutritional status. We suggested much more conservative opinions on uncut Roux-en-Y reconstruction.

## Supplementary Information

Below is the link to the electronic supplementary material.

Supplementary file1 (MP4 1835 KB)

Supplementary file2 (MP4 14697 KB)

Supplementary file3 (DOCX 21 KB)

Supplementary file4 (DOCX 31 KB)

Supplementary file5 (DOCX 158 KB)

Supplementary file6 (DOCX 54 KB)
