# Supplementary material for: To cut or not to cut? A prospective randomized controlled trial on short-term outcomes of the uncut Roux-en-Y reconstruction for gastric cancer
Source: Surg Endosc. 2023 May 9;37(8):6172–84. doi: 10.1007/s00464-023-10067-0 (PMC10338403; doi:10.1007/s00464-023-10067-0)
Supplement: Supplementary file 3 — Supplementary file3 (DOCX 21 KB) [file 464_2023_10067_MOESM3_ESM.docx]

**Uncut Roux-en-Y Gastrojejunostomy for Early Gastric Cancer Patients: a randomized controlled trial: trial protocol**

| **Title** | Uncut Roux-en-Y Gastrojejunostomy for Early Gastric Cancer Patients |
| --- | --- |
| **Study Site** | The First Affiliated Hospital with Nanjing Medical University, Nanjing, Jiangsu, China |
| **Study Period** | Recruitment start date: April 2016  Recruitment end date: October 2019  Follow-up period end date: October 2020  Final analysis date: December 2020 |
| **Population** | Patients with primary gastric cancer with a clinical stage I tumor according to the American Joint Committee on Cancer (AJCC-UICC) TNM classification for gastric cancer (7th edition) |
| **Endpoints** | **Primary Endpoint:**  The quality-of-life score measured by QLQ-STO22 up to 12 months after surgery  **Secondary endpoints:**  The clinical indicators during operation, early recovery course, postoperative complications, and nutritional status |
| **Trial design** | Prospective, open-label, phase II randomized controlled study |
| **Inclusion and exclusion criteria** | **Inclusion criteria：**  (1) aged 18–70 years  (2) bearing a distal gastric adenocarcinoma of >5 cm from the cardia confirmed by endoscopic biopsy and suitable for distal gastrectomy  (3) with a clinical stage I tumor  (4) devoid of any mental illness  (5) able to fill out the EORTC questionnaires  (6) having provided written consent  (7) available for followed-up until the end of the study  **Exclusion criteria:**  (1) necessitating chemotherapy pre- or post-surgery according to the NCCN guidelines to avoid interference with the detection of postoperative QOL differences  (2) pregnant or breastfeeding women  (3) bearing ongoing infections  (4) bearing severe mental disorders  (5) bearing serious cardiovascular diseases, liver or kidney dysfunction (glutamic-pyruvic or glutamic-oxaloacetic transaminases and serum creatinine of >300% and >150% higher than normal, respectively), abnormal blood clotting function (mean prothrombin and activated partial thromboplastin higher than the normal limit of 50%), neuropsychiatric disorders  (6) bearing other malignant tumors  (7) having requested to be excluded from the study  (8) having had total gastrectomy due to unsuitable for distal gastrectomy |
| **Study procedure** | **Randomization and Data Monitoring**  To exclude the influence of different surgeons on the results, an interactive web-based response system deploying a central, dynamic, and stratified randomization procedure was used to assign eligible patients. The stratification factors of the randomization process were surgeons at our center. The enrolled patients were divided into two groups, an uncut R-Y and a classic R-Y group, without prior knowledge. Our research center (The First Affiliated Hospital of Nanjing Medical University, Nanjing, China) was responsible for this study.  **Eligibility of Surgeons**  Surgeons who met the following criteria qualified for this surgery: (1) had performed at least 500 distal gastrectomies with D2 lymphadenectomy by open or laparoscopic approaches, (2) had performed at least 300 gastrectomies for patients with GC annually at our center, (3) had been trained strictly on GCP. All unedited video files of the surgical procedures involved in this trial were stored.  **Surgical technique**  Surgery was conducted as previously reported[^17^](#_ENREF_17). Under general anesthesia, the patient was placed in the supine position. The surgeon stood on the patient’s left, the first assistant on their right, and the camera operator between the patient’s legs. After establishing pneumoperitoneum, five ports were generated, and an electro-laparoscope was introduced through the umbilical port. A D2 lymph node dissection as defined in the Japanese Gastric Cancer Treatment Guidelines was performed in this clinical trial[^18^](#_ENREF_18).  *Uncut R-Y method*  After lymphadenectomy, specimens were removed from the abdominal cavity and confirmed with negative margins. Reconstruction was then initiated. The transverse colon was lifted to expose the ligament of Treitz, the jejunum at 20 cm distally to the ligament was marked with a thread and taken out, and an extracorporeal side-to-side anastomosis was generated. A stapler without a blade (Ethicon Endo-Surgery AKT45) was used to block the afferent jejunum. The small bowel was returned to the abdominal cavity and the pneumoperitoneum was reconstructed after closing the abdominal incision. The jejunum, 5 cm distally to the occlusive line, was anastomosed to the side of the residual stomach using a 60 mm linear stapler (Ethicon Endo-Surgery Echelon 60). Their common entry was then closed using another 60 mm linear stapler. A drainage tube was inserted after peritoneal irrigation.  *Classic R-Y method*  The difference between this and the previous procedure was that a linear stapler was used to block the afferent limbs. In the R-Y group, a normal linear stapler with a blade (Ethicon Endo-Surgery Echelon 60) was used.  **Outcome measurements and follow-up**  The nutritional status of the patients was determined by routine blood and blood-chemistry tests at POM 3, 6, 9, and 12. The clinical research coordinator at the data center communicated with the patient and help them fill EORTC QLQ-STO22 questionnaires. The surgeon reported the operation time, blood loss, lymph node retrieval, and pathological findings.  Endoscopic examinations were conducted at POM 6 and 12 according to the NCCN guidelines. For recanalization determination, endoscopy and gastrointestinal radiography results at POM 12 were used as guiding evidence. |
| **Principle investigator** | Zekuan Xu |
