# Supplementary material for: To cut or not to cut? A prospective randomized controlled trial on short-term outcomes of the uncut Roux-en-Y reconstruction for gastric cancer
Source: Surg Endosc. 2023 May 9;37(8):6172–84. doi: 10.1007/s00464-023-10067-0 (PMC10338403; doi:10.1007/s00464-023-10067-0)
Supplement: Supplementary file 4 — Supplementary file4 (DOCX 31 KB) [file 464_2023_10067_MOESM4_ESM.docx]

|  | **Patient ID** | **Group** | **Enrollment Date** | **Surgeon** |
| --- | --- | --- | --- | --- |
| 1 | 0773866 | cut | 2016.04.05 | A |
| 2 | 0862959 | cut | 2016.04.07 | A |
| 3 | 0864116 | cut | 2016.04.08 | B |
| 4 | 0863842 | uncut | 2016.04.12 | A |
| 5 | 0866067 | cut | 2016.04.21 | A |
| 6 | 0866061 | cut | 2016.04.21 | A |
| 7 | 0869738 | cut | 2016.05.17 | A |
| 8 | 0870290 | uncut | 2016.05.25 | A |
| 9 | 0871309 | cut | 2016.05.31 | A |
| 10 | 0871628 | uncut | 2016.05.31 | A |
| 11 | 0872523 | uncut | 2016.06.07 | A |
| 12 | 0872976 | uncut | 2016.06.14 | A |
| 13 | 0873275 | uncut | 2016.06.14 | A |
| 14 | 0874147 | uncut | 2016.06.21 | A |
| 15 | 0874684 | uncut | 2016.06.23 | A |
| 16 | 0875321 | uncut | 2016.06.28 | A |
| 17 | 0875534 | uncut | 2016.06.30 | A |
| 18 | 0876855 | uncut | 2016.07.13 | A |
| 19 | 0136884 | uncut | 2016.07.15 | A |
| 20 | 0877986 | uncut | 2016.07.18 | B |
| 21 | 0879622 | cut | 2016.07.29 | A |
| 22 | 0877890 | uncut | 2016.08.10 | A |
| 23 | 0882539 | uncut | 2016.08.19 | A |
| 24 | 0884811 | cut | 2016.09.06 | A |
| 25 | 0885922 | uncut | 2016.09.13 | A |
| 26 | 0889176 | uncut | 2016.10.09 | A |
| 27 | 0890064 | cut | 2016.10.18 | A |
| 28 | 046807 | cut | 2016.10.25 | A |
| 29 | 0891349 | cut | 2016.10.25 | A |
| 30 | 0891559 | cut | 2016.10.27 | A |
| 31 | 0892290 | cut | 2016.11.01 | A |
| 32 | 0893290 | uncut | 2016.11.08 | A |
| 33 | 0893357 | cut | 2016.11.09 | A |
| 34 | 0893861 | uncut | 2016.11.15 | A |
| 35 | 0893992 | cut | 2016.11.15 | A |
| 36 | 0895476 | uncut | 2016.11.29 | A |
| 37 | 0896560 | uncut | 2016.12.05 | A |
| 38 | 0893431 | uncut | 2016.12.13 | A |
| 39 | 0874541 | uncut | 2016.12.15 | A |
| 40 | 0900037 | uncut | 2017.01.03 | A |
| 41 | 0899484 | cut | 2017.01.03 | A |
| 42 | 0899026 | cut | 2017.01.05 | A |
| 43 | 0901680 | uncut | 2017.01.10 | A |
| 44 | 0898680 | uncut | 2017.01.10 | A |
| 45 | 0901946 | uncut | 2017.01.11 | A |
| 46 | 0901257 | uncut | 2017.01.11 | B |
| 47 | 0902811 | uncut | 2017.01.17 | A |
| 48 | 0902819 | uncut | 2017.01.18 | B |
| 49 | 0903849 | uncut | 2017.01.24 | A |
| 50 | 0801486 | uncut | 2017.02.08 | B |
| 51 | 0904318 | uncut | 2017.02.09 | A |
| 52 | 0906772 | uncut | 2017.02.21 | A |
| 53 | 0907087 | cut | 2017.02.21 | A |
| 54 | 0908319 | cut | 2017.03.08 | A |
| 55 | 0909538 | cut | 2017.03.14 | A |
| 56 | 0911729 | cut | 2017.04.02 | B |
| 57 | 0915734 | cut | 2017.04.25 | A |
| 58 | 0916474 | cut | 2017.05.02 | A |
| 59 | 0917212 | uncut | 2017.05.05 | A |
| 60 | 0919267 | cut | 2017.05.23 | A |
| 61 | 0920299 | cut | 2017.05.25 | A |
| 62 | 0920363 | uncut | 2017.05.31 | A |
| 63 | 0921405 | cut | 2017.06.06 | A |
| 64 | 0921625 | cut | 2017.06.06 | A |
| 65 | 0920316 | uncut | 2017.06.06 | A |
| 66 | 0921779 | uncut | 2017.06.07 | A |
| 67 | 0921423 | uncut | 2017.06.13 | B |
| 68 | 0925161 | uncut | 2017.06.28 | A |
| 69 | 0921858 | uncut | 2017.07.04 | A |
| 70 | 0926859 | uncut | 2017.07.14 | A |
| 71 | 0928025 | uncut | 2017.07.25 | A |
| 72 | 0928816 | uncut | 2017.07.25 | A |
| 73 | 0929044 | cut | 2017.07.27 | A |
| 74 | 0929771 | uncut | 2017.07.31 | B |
| 75 | 185809 | uncut | 2017.08.10 | A |
| 76 | 0932879 | uncut | 2017.08.22 | B |
| 77 | 0935068 | uncut | 2017.09.06 | B |
| 78 | 0933919 | uncut | 2017.09.14 | A |
| 79 | 0936748 | cut | 2017.09.19 | A |
| 80 | 0937262 | uncut | 2017.09.26 | B |
| 81 | 0938681 | cut | 2017.10.10 | A |
| 82 | 0939167 | uncut | 2017.10.10 | A |
| 83 | 0938961 | uncut | 2017.10.10 | A |
| 84 | 0938038 | cut | 2017.10.31 | A |
| 85 | 0739704 | uncut | 2017.11.10 | A |
| 86 | 0944356 | uncut | 2017.11.16 | A |
| 87 | 0946357 | uncut | 2017.11.29 | A |
| 88 | 0542122 | uncut | 2017.12.13 | A |
| 89 | 0923809 | cut | 2018.01.05 | A |
| 90 | 0577826 | cut | 2018.01.31 | A |
| 91 | 0963118 | cut | 2018.03.22 | A |
| 92 | 0964539 | uncut | 2018.03.30 | A |
| 93 | 0964235 | cut | 2018.03.30 | A |
| 94 | 0965969 | uncut | 2018.04.10 | A |
| 95 | 0967841 | cut | 2018.04.24 | A |
| 96 | 0967493 | cut | 2018.05.03 | A |
| 97 | 0971555 | uncut | 2018.05.10 | A |
| 98 | 0972070 | uncut | 2018.05.15 | A |
| 99 | 0972534 | cut | 2018.05.17 | A |
| 100 | 0971386 | cut | 2018.05.22 | A |
| 101 | 0976100 | uncut | 2018.06.05 | A |
| 102 | 0977972 | uncut | 2018.06.19 | A |
| 103 | 0978396 | uncut | 2018.06.20 | A |
| 104 | 0981719 | cut | 2018.07.10 | A |
| 105 | 0983798 | cut | 2018.07.24 | A |
| 106 | 0984244 | uncut | 2018.07.26 | A |
| 107 | 0985072 | uncut | 2018.07.27 | A |
| 108 | 0985667 | cut | 2018.07.31 | A |
| 109 | 0992717 | cut | 2018.09.06 | A |
| 110 | 0993413 | cut | 2018.09.11 | A |
| 111 | 0918878 | uncut | 2018.09.18 | A |
| 112 | 0994641 | cut | 2018.09.18 | A |
| 113 | 0994783 | cut | 2018.09.20 | A |
| 114 | 0999878 | uncut | 2018.10.23 | A |
| 115 | 1000057 | cut | 2018.10.23 | A |
| 116 | 1001611 | cut | 2018.10.30 | B |
| 117 | 1005471 | cut | 2018.11.20 | B |
| 118 | 1006340 | cut | 2018.11.21 | A |
| 119 | 1008408 | cut | 2018.12.04 | A |
| 120 | 1007607 | uncut | 2018.12.04 | A |
| 121 | 1011023 | uncut | 2018.12.18 | A |
| 122 | 1016942 | cut | 2019.01.15 | A |
| 123 | 1017278 | uncut | 2019.01.17 | A |
| 124 | 1017118 | uncut | 2019.01.17 | A |
| 125 | 1017743 | uncut | 2019.01.22 | A |
| 126 | 1018898 | uncut | 2019.01.24 | B |
| 127 | 1024347 | cut | 2019.03.06 | B |
| 128 | 1030086 | uncut | 2019.03.26 | A |
| 129 | 1030530 | cut | 2019.03.28 | A |
| 130 | 1030936 | cut | 2019.03.28 | A |
| 131 | 1033379 | uncut | 2019.04.10 | A |
| 132 | 1033994 | cut | 2019.04.12 | A |
| 133 | 1040893 | cut | 2019.05.21 | A |
| 134 | 1043264 | uncut | 2019.05.28 | A |
| 135 | 1043728 | cut | 2019.05.28 | A |
| 136 | 1044859 | cut | 2019.06.04 | A |
| 137 | 1044474 | cut | 2019.06.04 | A |
| 138 | 1043745 | uncut | 2019.06.05 | A |
| 139 | 1046795 | cut | 2019.06.11 | A |
| 140 | 1044515 | uncut | 2019.06.11 | A |
| 141 | 1046966 | cut | 2019.06.12 | A |
| 142 | 1046965 | uncut | 2019.06.12 | A |
| 143 | 1050377 | cut | 2019.06.27 | B |
| 144 | 1051072 | cut | 2019.07.02 | A |
| 145 | 1051360 | cut | 2019.07.04 | A |
| 146 | 1051709 | cut | 2019.07.04 | A |
| 147 | 1052408 | cut | 2019.07.09 | A |
| 148 | 1054287 | cut | 2019.07.16 | A |
| 149 | 1053829 | cut | 2019.07.18 | A |
| 150 | 1055246 | cut | 2019.07.23 | A |
| 151 | 1055968 | cut | 2019.07.25 | B |
| 152 | 0823517 | cut | 2019.08.06 | A |
| 153 | 1058545 | uncut | 2019.08.06 | A |
| 154 | 1058607 | cut | 2019.08.06 | B |
| 155 | 1059273 | uncut | 2019.08.08 | A |
| 156 | 1060519 | uncut | 2019.08.14 | A |
| 157 | 1059974 | cut | 2019.08.14 | B |
| 158 | 1054893 | cut | 2019.08.15 | A |
| 159 | 1063068 | uncut | 2019.08.28 | A |
| 160 | 1063734 | cut | 2019.08.29 | A |
| 161 | 1065756 | cut | 2019.09.10 | A |
| 162 | 1066465 | cut | 2019.09.10 | A |
| 163 | 1066767 | cut | 2019.09.12 | A |
| 164 | 1067798 | cut | 2019.09.17 | A |
| 165 | 1068451 | cut | 2019.09.24 | A |
| 166 | 1072677 | uncut | 2019.10.15 | A |
| 167 | 1075342 | cut | 2019.10.25 | A |
| 168 | 1075488 | cut | 2019.10.28 | B |
| 169 | 1075863 | cut | 2019.10.30 | A |
| 170 | 1076145 | uncut | 2019.10.30 | A |
