# Supplementary figures and images for: To cut or not to cut? A prospective randomized controlled trial on short-term outcomes of the uncut Roux-en-Y reconstruction for gastric cancer
Source: Surg Endosc. 2023 May 9;37(8):6172–84. doi: 10.1007/s00464-023-10067-0 (PMC10338403; doi:10.1007/s00464-023-10067-0)

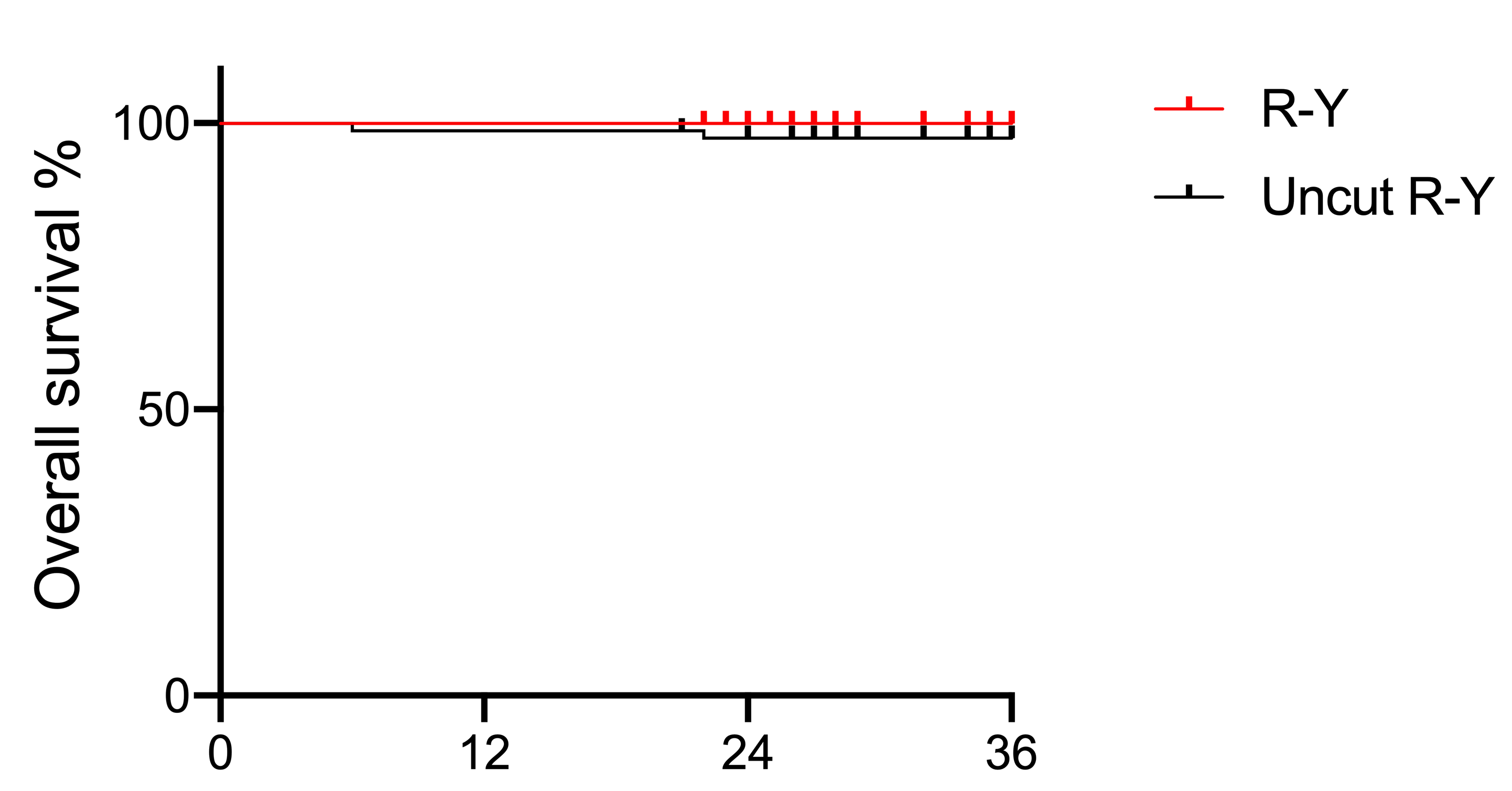


Supplementary Figure S1 Overall survival analysis of patients in R-Y group and Uncut R-Y group

Supplement: Supplementary file 5 — Supplementary file5 (DOCX 158 KB) [file 464_2023_10067_MOESM5_ESM.docx]
