# Supplementary material for: To cut or not to cut? A prospective randomized controlled trial on short-term outcomes of the uncut Roux-en-Y reconstruction for gastric cancer
Source: Surg Endosc. 2023 May 9;37(8):6172–84. doi: 10.1007/s00464-023-10067-0 (PMC10338403; doi:10.1007/s00464-023-10067-0)
Supplement: Supplementary file 6 — Supplementary file6 (DOCX 54 KB) [file 464_2023_10067_MOESM6_ESM.docx]

|  | R-Y group  (n=72) | Uncut R-Y group  (n=76) | *p* value |
| --- | --- | --- | --- |
| **Dysphagia scale** |  |  |  |
| 3^rd^ | 1.11±0.20 | 1.10±0.18 | 0.736 |
| 6^th^ | 1.11±0.18 | 1.13±0.20 | 0.747 |
| 9^th^ | 1.12±0.21 | 1.09±0.19 | 0.341 |
| 12^th^ | 1.06±0.15 | 1.05±0.15 | 0.491 |
| **Pain scale** |  |  |  |
| 3^rd^ | 1.22±0.22 | 1.24±0.30 | 0.691 |
| 6^th^ | 1.20±0.24 | 1.19±0.26 | 0.757 |
| 9^th^ | 1.12±0.20 | 1.18±0.28 | 0.154 |
| 12^th^ | 1.12±0.20 | 1.19±0.29 | 0.089 |
| **Reflux symptoms scale** |  |  |  |
| 3^rd^ | 1.22±0.23 | 1.29±0.36 | 0.241 |
| 6^th^ | 1.18±0.22 | 1.21±0.26 | 0.404 |
| 9^th^ | 1.14±0.20 | 1.23±0.27 | 0.029* |
| 12^th^ | 1.13±0.24 | 1.11±0.17 | 0.575 |
| **Eating restriction scale** |  |  |  |
| 3^rd^ | 1.08±0.17 | 1.14±0.23 | 0.118 |
| 6^th^ | 1.10±0.17 | 1.15±0.23 | 0.181 |
| 9^th^ | 1.08±0.16 | 1.23±0.39 | 0.004** |
| 12^th^ | 1.08±0.15 | 1.15±0.24 | 0.045* |
| **Anxiety scale** |  |  |  |
| 3^rd^ | 1.34±0.43 | 1.38±0.51 | 0.674 |
| 6^th^ | 1.27±0.33 | 1.26±0.32 | 0.955 |
| 9^th^ | 1.25±0.44 | 1.38±0.54 | 0.152 |
| 12^th^ | 1.18±0.36 | 1.33±0.42 | 0.022* |
| **Hair loss** |  |  |  |
| 3^rd^ | 1.18±0.38 | 1.11±0.21 | 0.192 |
| 6^th^ | 1.13±0.33 | 1.06±0.18 | 0.166 |
| 9^th^ | 1.10±0.26 | 1.17±0.42 | 0.204 |
| 12^th^ | 1.04±0.23 | 1.04±0.32 | 0.879 |

Supplementary Table 1. Detailed data of Quality of life accessed by QLQ-STO22 questionnaires between R-Y and uncut R-Y groups. * *p*<0.05, ***p*<0.01, ****p*<0.001.

|  | R-Y group  (n=72) | Uncut R-Y without recanalization (n=49) | *p* value |
| --- | --- | --- | --- |
| **Dysphagia scale** |  |  |  |
| 3^rd^ | 1.11±0.20 | 1.10±0.17 | 0.693 |
| 6^th^ | 1.12±0.18 | 1.14±0.23 | 0.475 |
| 9^th^ | 1.12±0.21 | 1.09±0.18 | 0.409 |
| 12^th^ | 1.06±0.15 | 1.04±0.11 | 0.351 |
| **Pain scale** |  |  |  |
| 3^rd^ | 1.23±0.23 | 1.16±0.25 | 0.186 |
| 6^th^ | 1.20±0.24 | 1.18±0.27 | 0.597 |
| 9^th^ | 1.17±0.21 | 1.19±0.25 | 0.622 |
| 12^th^ | 1.12±0.20 | 1.17±0.31 | 0.260 |
| **Reflux symptoms scale** |  |  |  |
| 3^rd^ | 1.23±0.23 | 1.31±0.40 | 0.173 |
| 6^th^ | 1.17±0.22 | 1.24±0.26 | 0.145 |
| 9^th^ | 1.14±0.20 | 1.22±0.30 | 0.102 |
| 12^th^ | 1.13±0.24 | 1.11±0.16 | 0.637 |
| **Eating restriction scale** |  |  |  |
| 3^rd^ | 1.08±0.17 | 1.11±0.20 | 0.421 |
| 6^th^ | 1.09±0.17 | 1.17±0.24 | 0.071 |
| 9^th^ | 1.09±0.17 | 1.26±0.43 | 0.004** |
| 12^th^ | 1.07±0.13 | 1.17±0.27 | 0.009** |
| **Anxiety scale** |  |  |  |
| 3^rd^ | 1.34±0.43 | 1.33±0.42 | 0.907 |
| 6^th^ | 1.26±0.33 | 1.28±0.33 | 0.781 |
| 9^th^ | 1.27±0.44 | 1.36±0.55 | 0.320 |
| 12^th^ | 1.18±0.36 | 1.36±0.41 | 0.013* |
| Hair loss |  |  |  |
| 3^rd^ | 1.17±0.38 | 1.11±0.21 | 0.410 |
| 6^th^ | 1.13±0.34 | 1.08±0.22 | 0.405 |
| 9^th^ | 1.10±0.26 | 1.17±0.32 | 0.181 |
| 12^th^ | 1.04±0.23 | 1.07±0.40 | 0.591 |

Supplementary Table 2. Detailed data of Quality of life accessed by QLQ-STO22 questionnaires between R-Y and uncut R-Y without recanalization group. * *p*<0.05, ***p*<0.01, ****p*<0.001

|  | R-Y group  (n=72) | Uncut R-Y group  (n=76) | *p* value |
| --- | --- | --- | --- |
| Hb (g/L) | 132.83±15.19 | 131.77±15.63 | 0.688 |
| RBC (×10^12^/L) | 4.36±0.47 | 4.44±0.62 | 0.380 |
| WBC (×10^9^/L) | 5.71±1.83 | 6.58±6.30 | 0.263 |
| Neu (×10^9^/L) | 3.52±1.87 | 4.45±6.52 | 0.245 |
| Lym (×10^9^/L) | 1.74±0.73 | 2.10±3.77 | 0.425 |
| PLT (×10^9^/L ) | 194.11±60.04 | 192.13±62.28 | 0.844 |
| ALT (U/L) | 20.50±12.02 | 20.72±12.89 | 0.958 |
| AST (U/L) | 21.94±8.87 | 22.60±8.06 | 0.689 |
| TBIL (umol/L) | 11.71±4.12 | 14.94±21.32 | 0.208 |
| DBIL (umol/L) | 3.80±1.54 | 4.02±1.85 | 0.438 |
| IBIL (umol/L) | 7.91±2.82 | 8.64±3.51 | 0.168 |
| BUN (mmol/L) | 5.82±1.48 | 5.64±1.38 | 0.441 |
| CRE (umol/L) | 72.75±44.93 | 72.91±41.38 | 0.995 |
| K (mmol/L) | 4.41±4.21 | 3.92±0.40 | 0.321 |
| Na (mmol/L) | 140.56±1.97 | 140.11±2.14 | 0.181 |
| Cl (mmol/L) | 105.41±2.46 | 105.00±2.40 | 0.345 |
| Ca (mmol/L) | 2.59±2.42 | 2.29±0.13 | 0.284 |
| Mg (mmol/L) | 0.88±0.13 | 0.89±0.08 | 0.382 |
| GLU (mmol/L) | 5.20±1.24 | 5.22±0.95 | 0.910 |
| UA (umol/L) | 304.20±90.60 | 278.19±86.31 | 0.076 |
| TP (g/L) | 66.04±7.16 | 66.33±5.72 | 0.791 |
| ALB (g/L) | 40.70±4.11 | 40.81±3.68 | 0.863 |
| GLO (g/L) | 25.78±4.15 | 25.16±5.15 | 0.422 |
| CEA (ng/ml) | 2.12±1.77 | 2.32±1.51 | 0.374 |
| CA199 (kU/L) | 9.39±4.72 | 9.99±6.56 | 0.679 |
| AFP (ng/ml) | 3.15±1.87 | 2.87±1.58 | 0.310 |
| CA724 (kU/L) | 7.42±4.96 | 5.17±12.69 | 0.613 |

Supplementary Table 3 Results of blood routine, blood chemistry, and tumor marker between R-Y group and Uncut R-Y group before surgery. **p*<0.05, ***p*<0.01, ****p*<0.001.

|  | R-Y group  (n=72) | Uncut R-Y group  (n=76) | *p* value |
| --- | --- | --- | --- |
| Hb (g/L) | 127.11±12.82 | 129.04±12.80 | 0.413 |
| RBC (×10^12^/L) | 4.25±0.46 | 4.29±0.39 | 0.581 |
| WBC (×10^9^/L) | 5.25±1.18 | 5.57±1.56 | 0.200 |
| Neu (×10^9^/L) | 3.65±6.05 | 3.32±1.41 | 0.690 |
| Lym (×10^9^/L) | 1.79±0.58 | 2.41±4.87 | 0.316 |
| PLT (×10^9^/L ) | 196.65±65.59 | 193.65±56.41 | 0.789 |
| ALT (U/L) | 24.86±14.77 | 21.22±12.46 | 0.147 |
| AST (U/L) | 24.34±9.19 | 22.36±6.40 | 0.174 |
| TBIL (umol/L) | 11.70±5.77 | 12.61±5.25 | 0.374 |
| DBIL (umol/L) | 4.38±2.53 | 4.48±1.83 | 0.803 |
| IBIL (umol/L) | 7.32±3.84 | 8.18±3.75 | 0.226 |
| BUN (mmol/L) | 5.35±1.44 | 5.45±1.26 | 0.713 |
| CRE (umol/L) | 64.66±19.50 | 63.43±13.44 | 0.692 |
| K (mmol/L) | 4.15±0.38 | 4.11±0.39 | 0.751 |
| Na (mmol/L) | 140.51±1.93 | 140.36±2.03 | 0.708 |
| Cl (mmol/L) | 104.19±2.31 | 103.92±2.23 | 0.523 |
| Ca (mmol/L) | 2.35±0.13 | 2.33±0.16 | 0.457 |
| Mg (mmol/L) | 0.88±0.07 | 0.94±0.11 | 0.002** |
| GLU (mmol/L) | 5.55±1.08 | 5.51±0.68 | 0.816 |
| UA (umol/L) | 329.73±272.27 | 282.44±79.32 | 0.220 |
| TP (g/L) | 68.69±6.72 | 69.84±4.42 | 0.277 |
| ALB (g/L) | 41.47±4.52 | 42.14±4.07 | 0.394 |
| GLO (g/L) | 27.09±4.57 | 27.54±3.75 | 0.566 |
| CEA (ng/ml) | 2.18±2.13 | 2.39±1.55 | 0.556 |
| CA199 (kU/L) | 8.71±5.28 | 8.50±6.44 | 0.846 |
| AFP (ng/ml) | 3.43±2.58 | 2.93±1.80 | 0.253 |
| CA724 (kU/L) | 3.10±4.76 | 3.68±6.94 | 0.711 |

Supplementary Table 4 Results of blood routine, blood chemistry, and tumor marker between R-Y group and Uncut R-Y group at 3^rd^ month after surgery. **p*<0.05, ***p*<0.01, ****p*<0.001.

|  | R-Y group  (n=72) | Uncut R-Y group  (n=76) | *p* value |
| --- | --- | --- | --- |
| Hb (g/L) | 127.28±14.43 | 130.4±12.54 | 0.228 |
| RBC (×10^12^/L) | 4.16±0.50 | 4.21±0.46 | 0.358 |
| WBC (×10^9^/L) | 5.40±1.45 | 5.15±1.49 | 0.369 |
| Neu (×10^9^/L) | 3.07±1.16 | 2.95±1.33 | 0.616 |
| Lym (×10^9^/L) | 1.82±0.57 | 1.76±0.53 | 0.547 |
| PLT (×10^9^/L ) | 200.75±52.98 | 180.56±47.46 | 0.037* |
| ALT (U/L) | 23.69±15.25 | 27.02±32.03 | 0.472 |
| AST (U/L) | 24.18±7.55 | 29.30±30.86 | 0.217 |
| TBIL (umol/L) | 11.59±4.39 | 13.01±5.06 | 0.116 |
| DBIL (umol/L) | 4.20±2.05 | 4.54±1.88 | 0.363 |
| IBIL (umol/L) | 7.27±3.12 | 8.47±3.67 | 0.066 |
| BUN (mmol/L) | 5.77±3.63 | 6.59±6.90 | 0.446 |
| CRE (umol/L) | 73.78±82.82 | 67.57±34.13 | 0.618 |
| K (mmol/L) | 4.15±0.40 | 4.09±0.57 | 0.651 |
| Na (mmol/L) | 140.71±2.04 | 139.96±2.32 | 0.103 |
| Cl (mmol/L) | 104.61±2.07 | 104.48±2.62 | 0.769 |
| Ca (mmol/L) | 2.36±0.11 | 2.34±0.18 | 0.399 |
| Mg (mmol/L) | 0.92±0.09 | 0.95±0.18 | 0.313 |
| GLU (mmol/L) | 5.65±1.20 | 5.57±0.99 | 0.685 |
| UA (umol/L) | 291.38±89.29 | 273.48±73.26 | 0.258 |
| TP (g/L) | 71.17±5.87 | 70.66±4.52 | 0.610 |
| ALB (g/L) | 43.15±3.64 | 43.61±4.05 | 0.524 |
| GLO (g/L) | 28.05±3.85 | 27.05±3.65 | 0.171 |
| CEA (ng/ml) | 2.35±1.49 | 2.54±1.67 | 0.462 |
| CA199 (kU/L) | 10.48±8.37 | 10.56±8.86 | 0.977 |
| AFP (ng/ml) | 3.51±2.54 | 2.67±1.75 | 0.059 |
| CA724 (kU/L) | 2.87±4.01 | 4.64±9.85 | 0.342 |

Supplementary Table 5 Results of blood routine, blood chemistry, and tumor marker between R-Y group and Uncut R-Y group at 6^th^ month after surgery. **p*<0.05, ***p*<0.01, ****p*<0.001.

|  | R-Y group  (n=72) | Uncut R-Y group  (n=76) | *p* value |
| --- | --- | --- | --- |
| Hb (g/L) | 130.03±12.88 | 133.57±12.93 | 0.228 |
| RBC (×10^12^/L) | 4.12±0.44 | 4.31±0.53 | 0.098 |
| WBC (×10^9^/L) | 5.29±1.39 | 5.42±1.57 | 0.695 |
| Neu (×10^9^/L) | 3.14±1.32 | 3.18±1.42 | 0.896 |
| Lym (×10^9^/L) | 1.69±0.57 | 1.90±0.61 | 0.126 |
| PLT (×10^9^/L ) | 199.82±60.81 | 175.12±50.11 | 0.046* |
| ALT (U/L) | 20.42±9.43 | 19.02±9.76 | 0.520 |
| AST (U/L) | 23.18±7.44 | 22.20±5.16 | 0.480 |
| TBIL (umol/L) | 11.49±5.13 | 12.64±4.99 | 0.315 |
| DBIL (umol/L) | 4.04±2.66 | 4.16±1.69 | 0.803 |
| IBIL (umol/L) | 7.30±3.39 | 8.48±3.62 | 0.142 |
| BUN (mmol/L) | 5.35±1.14 | 5.47±1.03 | 0.637 |
| CRE (umol/L) | 64.10±12.64 | 62.7±12.17 | 0.617 |
| K (mmol/L) | 4.08±0.42 | 4.11±0.64 | 0.787 |
| Na (mmol/L) | 140.48±2.50 | 139.95±2.21 | 0.347 |
| Cl (mmol/L) | 103.79±1.92 | 103.97±2.64 | 0.748 |
| Ca (mmol/L) | 2.31±0.13 | 2.31±0.20 | 0.912 |
| Mg (mmol/L) | 0.90±0.07 | 0.90±0.08 | 0.955 |
| GLU (mmol/L) | 5.63±1.32 | 5.43±0.62 | 0.353 |
| UA (umol/L) | 280.18±60.75 | 281.45±66.72 | 0.931 |
| TP (g/L) | 68.90±5.25 | 71.86±4.53 | 0.008** |
| ALB (g/L) | 42.22±4.28 | 43.20±3.12 | 0.233 |
| GLO (g/L) | 26.68±3.68 | 28.67±3.83 | 0.022* |
| CEA (ng/ml) | 2.28±1.34 | 2.37±1.54 | 0.887 |
| CA199 (kU/L) | 9.63±5.16 | 11.45±11.57 | 0.389 |
| AFP (ng/ml) | 2.75±1.40 | 2.71±1.86 | 0.916 |
| CA724 (kU/L) | 2.77±4.34 | 4.02±8.53 | 0.579 |

Supplementary Table 6 Results of blood routine, blood chemistry, and tumor marker between R-Y group and Uncut R-Y group at 9^th^ month after surgery. **p*<0.05, ***p*<0.01, ****p*<0.001.

|  | R-Y group  (n=72) | Uncut R-Y group  (n=76) | *p* value |
| --- | --- | --- | --- |
| Hb (g/L) | 127.66±20.51 | 130.13±16.67 | 0.596 |
| RBC (×10^12^/L) | 4.21±0.43 | 4.32±0.47 | 0.201 |
| WBC (×10^9^/L) | 5.59±1.32 | 5.56±1.48 | 0.889 |
| Neu (×10^9^/L) | 3.28±1.22 | 3.24±1.24 | 0.863 |
| Lym (×10^9^/L) | 1.72±0.55 | 1.74±0.55 | 0.873 |
| PLT (×10^9^/L ) | 187.89±39.53 | 182.61±49.05 | 0.441 |
| ALT (U/L) | 25.84±23.47 | 19.92±8.75 | 0.171 |
| AST (U/L) | 26.92±16.03 | 23.80±7.25 | 0.181 |
| TBIL (umol/L) | 11.66±3.80 | 12.72±6.32 | 0.287 |
| DBIL (umol/L) | 4.31±1.76 | 4.49±2.18 | 0.644 |
| IBIL (umol/L) | 7.34±2.37 | 8.15±4.26 | 0.227 |
| BUN (mmol/L) | 5.70±1.08 | 5.71±1.29 | 0.969 |
| CRE (umol/L) | 63.71±12.63 | 64.5±13.00 | 0.743 |
| K (mmol/L) | 4.11±0.55 | 4.29±0.34 | 0.045* |
| Na (mmol/L) | 140.83±1.96 | 141.01±2.26 | 0.663 |
| Cl (mmol/L) | 104.66±1.80 | 104.35±2.28 | 0.447 |
| Ca (mmol/L) | 2.34±0.10 | 2.33±0.13 | 0.884 |
| Mg (mmol/L) | 0.88±0.08 | 0.91±0.10 | 0.089 |
| GLU (mmol/L) | 5.60±1.43 | 5.46±0.73 | 0.508 |
| UA (umol/L) | 295.79±58.28 | 274.49±64.75 | 0.071 |
| TP (g/L) | 71.07±4.90 | 70.39±6.54 | 0.542 |
| ALB (g/L) | 43.04±3.28 | 42.36±4.62 | 0.338 |
| GLO (g/L) | 27.95±4.28 | 27.74±5.23 | 0.809 |
| CEA (ng/ml) | 2.54±1.55 | 2.83±1.64 | 0.380 |
| CA199 (kU/L) | 10.09±4.83 | 10.82±8.54 | 0.496 |
| AFP (ng/ml) | 3.25±2.78 | 2.66±1.69 | 0.179 |
| CA724 (kU/L) | 2.33±2.01 | 1.87±1.67 | 0.176 |

Supplementary Table 7 Results of blood routine, blood chemistry, and tumor marker between R-Y group and Uncut R-Y group at 12^th^ month after surgery. **p*<0.05, ***p*<0.01, ****p*<0.001.

|  | R-Y group  (n=72) | Uncut R-Y without recanalization (n=49) | *p* value |
| --- | --- | --- | --- |
| Hb (g/L) | 132.83±15.19 | 133.50±14.84 | 0.802 |
| RBC (×10^12^/L) | 4.36±0.47 | 4.55±0.66 | 0.067 |
| WBC (×10^9^/L) | 5.71±1.83 | 7.06±7.48 | 0.144 |
| Neu (×10^9^/L) | 3.52±1.87 | 4.94±7.74 | 0.136 |
| Lym (×10^9^/L) | 1.74±0.73 | 2.32±4.68 | 0.306 |
| PLT (×10^9^/L ) | 194.11±60.04 | 189.55±63.21 | 0.689 |
| ALT (U/L) | 20.5±12.02 | 20.24±12.65 | 0.854 |
| AST (U/L) | 21.94±8.87 | 22.24±8.37 | 0.849 |
| TBIL (umol/L) | 11.71±4.12 | 17.00±26.24 | 0.095 |
| DBIL (umol/L) | 3.80±1.54 | 4.30±1.94 | 0.119 |
| IBIL (umol/L) | 7.91±2.82 | 9.15±3.76 | 0.040* |
| BUN (mmol/L) | 5.82±1.48 | 5.63±1.53 | 0.495 |
| CRE (umol/L) | 72.75±44.93 | 78.62±49.97 | 0.508 |
| K (mmol/L) | 4.41±4.21 | 3.96±0.45 | 0.461 |
| Na (mmol/L) | 140.56±1.97 | 140.14±2.14 | 0.268 |
| Cl (mmol/L) | 105.41±2.46 | 104.75±2.32 | 0.241 |
| Ca (mmol/L) | 2.59±2.42 | 2.30±0.12 | 0.404 |
| Mg (mmol/L) | 0.88±0.13 | 0.89±0.08 | 0.589 |
| GLU (mmol/L) | 5.20±1.24 | 5.14±1.00 | 0.780 |
| UA (umol/L) | 304.20±90.60 | 289.16±87.59 | 0.366 |
| TP (g/L) | 66.04±7.16 | 66.32±5.29 | 0.819 |
| ALB (g/L) | 40.70±4.11 | 41.03±3.66 | 0.643 |
| GLO (g/L) | 25.78±4.15 | 25.18±4.24 | 0.439 |
| CEA (ng/ml) | 2.12±1.77 | 2.50±1.56 | 0.167 |
| CA199 (kU/L) | 9.26±4.80 | 10.14±6.77 | 0.382 |
| AFP (ng/ml) | 3.15±1.87 | 2.74±1.49 | 0.200 |
| CA724 (kU/L) | 7.42±36.61 | 6.52±15.36 | 0.665 |

Supplementary Table 8 Results of blood routine, blood chemistry, and tumor marker between R-Y group and Uncut R-Y without recanalization group before surgery. **p*<0.05, ***p*<0.01, ****p*<0.001.

|  | R-Y group  (n=72) | Uncut R-Y without recanalization (n=49) | *p* value |
| --- | --- | --- | --- |
| Hb (g/L) | 127.11±12.82 | 130.31±12.65 | 0.639 |
| RBC (×10^12^/L) | 4.25±0.46 | 4.35±0.36 | 0.254 |
| WBC (×10^9^/L) | 5.25±1.18 | 5.91±1.65 | 0.026* |
| Neu (×10^9^/L) | 3.65±6.05 | 3.67±1.49 | 0.985 |
| Lym (×10^9^/L) | 1.79±0.58 | 2.84±6.49 | 0.197 |
| PLT (×10^9^/L ) | 196.65±65.59 | 199.13±66.44 | 0.862 |
| ALT (U/L) | 24.86±14.77 | 20.01±10.06 | 0.098 |
| AST (U/L) | 24.34±9.19 | 21.6±4.80 | 0.116 |
| TBIL (umol/L) | 11.70±5.77 | 12.77±5.66 | 0.392 |
| DBIL (umol/L) | 4.38±2.53 | 4.58±1.96 | 0.701 |
| IBIL (umol/L) | 7.32±3.84 | 8.30±4.02 | 0.261 |
| BUN (mmol/L) | 5.35±1.44 | 5.46±1.19 | 0.710 |
| CRE (umol/L) | 64.66±19.50 | 65.38±15.36 | 0.857 |
| K (mmol/L) | 4.15±0.38 | 4.18±0.38 | 0.597 |
| Na (mmol/L) | 140.51±1.93 | 140.39±2.41 | 0.806 |
| Cl (mmol/L) | 104.19±2.31 | 104.09±2.56 | 0.845 |
| Ca (mmol/L) | 2.35±0.13 | 2.32±0.19 | 0.347 |
| Mg (mmol/L) | 0.88±0.07 | 0.93±0.14 | 0.018* |
| GLU (mmol/L) | 5.55±1.08 | 5.47±0.71 | 0.691 |
| UA (umol/L) | 329.73±272.27 | 297.10±80.75 | 0.510 |
| TP (g/L) | 68.69±6.72 | 69.71±5.00 | 0.450 |
| ALB (g/L) | 41.47±4.52 | 41.77±4.43 | 0.757 |
| GLO (g/L) | 27.09±4.57 | 27.67±4.20 | 0.558 |
| CEA (ng/ml) | 2.18±2.13 | 2.81±1.81 | 0.167 |
| CA199 (kU/L) | 8.71±5.28 | 8.26±6.63 | 0.724 |
| AFP (ng/ml) | 3.43±2.58 | 2.72±1.55 | 0.179 |
| CA724 (kU/L) | 11.83±51.13 | 5.07±10.03 | 0.682 |

Supplementary Table 9 Results of blood routine, blood chemistry, and tumor marker between R-Y group and Uncut R-Y without recanalization group at 3^rd^ month after surgery. **p*<0.05, ***p*<0.01, ****p*<0.001.

|  | R-Y group  (n=72) | Uncut R-Y without recanalization (n=49) | *p* value |
| --- | --- | --- | --- |
| Hb (g/L) | 127.28±14.43 | 133.24±11.83 | 0.747 |
| RBC (×10^12^/L) | 4.16±0.50 | 4.27±0.41 | 0.498 |
| WBC (×10^9^/L) | 5.40±1.45 | 5.47±1.54 | 0.834 |
| Neu (×10^9^/L) | 3.07±1.16 | 3.29±1.45 | 0.441 |
| Lym (×10^9^/L) | 1.82±0.57 | 1.73±0.54 | 0.468 |
| PLT (×10^9^/L ) | 200.75±52.98 | 178.48±49.08 | 0.061 |
| ALT (U/L) | 23.69±15.25 | 28.20±39.64 | 0.510 |
| AST (U/L) | 24.18±7.55 | 30.81±40.25 | 0.220 |
| TBIL (umol/L) | 11.59±4.39 | 12.46±4.42 | 0.392 |
| DBIL (umol/L) | 4.20±2.05 | 4.09±1.45 | 0.803 |
| IBIL (umol/L) | 7.27±3.12 | 8.36±3.63 | 0.150 |
| BUN (mmol/L) | 5.77±3.63 | 7.34±9.11 | 0.272 |
| CRE (umol/L) | 73.78±82.82 | 72.64±44.78 | 0.946 |
| K (mmol/L) | 4.15±0.40 | 4.03±0.72 | 0.420 |
| Na (mmol/L) | 140.71±2.04 | 139.62±2.40 | 0.050 |
| Cl (mmol/L) | 104.61±2.07 | 104.69±2.87 | 0.894 |
| Ca (mmol/L) | 2.36±0.11 | 2.32±0.23 | 0.250 |
| Mg (mmol/L) | 0.92±0.09 | 0.95±0.15 | 0.302 |
| GLU (mmol/L) | 5.65±1.20 | 5.49±0.99 | 0.531 |
| UA (umol/L) | 291.38±89.29 | 279.93±83.10 | 0.570 |
| TP (g/L) | 71.17±5.87 | 70.83±4.35 | 0.783 |
| ALB (g/L) | 43.15±3.64 | 44.05±4.36 | 0.309 |
| GLO (g/L) | 28.05±3.85 | 26.79±3.52 | 0.148 |
| CEA (ng/ml) | 2.32±1.50 | 2.81±2.05 | 0.213 |
| CA199 (kU/L) | 10.32±8.40 | 10.34±5.20 | 0.990 |
| AFP (ng/ml) | 3.51±2.54 | 2.51±1.79 | 0.083 |
| CA724 (kU/L) | 2.87±4.01 | 5.41±11.54 | 0.255 |

Supplementary Table 10 Results of blood routine, blood chemistry, and tumor marker between R-Y group and Uncut R-Y without recanalization group at 6^th^ month after surgery. **p*<0.05, ***p*<0.01, ****p*<0.001.

|  | R-Y group  (n=72) | Uncut R-Y without recanalization (n=49) | *p* value |
| --- | --- | --- | --- |
| Hb (g/L) | 130.03±12.88 | 136.97±13.33 | 0.042* |
| RBC (×10^12^/L) | 4.12±0.44 | 4.42±0.51 | 0.014* |
| WBC (×10^9^/L) | 5.29±1.39 | 5.68±1.85 | 0.343 |
| Neu (×10^9^/L) | 3.14±1.33 | 3.52±1.72 | 0.333 |
| Lym (×10^9^/L) | 1.69±0.57 | 1.91±0.61 | 0.157 |
| PLT (×10^9^/L ) | 199.82±60.81 | 172.27±53.07 | 0.061 |
| ALT (U/L) | 20.42±9.43 | 19.12±11.35 | 0.621 |
| AST (U/L) | 23.18±7.44 | 21.71±4.89 | 0.361 |
| TBIL (umol/L) | 11.49±5.13 | 12.52±5.02 | 0.422 |
| DBIL (umol/L) | 4.04±2.66 | 3.85±1.67 | 0.744 |
| IBIL (umol/L) | 7.30±3.39 | 8.67±3.68 | 0.129 |
| BUN (mmol/L) | 5.35±1.14 | 5.64±1.09 | 0.341 |
| CRE (umol/L) | 64.10±12.64 | 64.47±13.86 | 0.911 |
| K (mmol/L) | 4.08±0.42 | 4.07±0.78 | 0.946 |
| Na (mmol/L) | 140.48±2.50 | 140.03±1.85 | 0.470 |
| Cl (mmol/L) | 103.79±1.92 | 104.88±2.73 | 0.092 |
| Ca (mmol/L) | 2.31±0.13 | 2.30±0.25 | 0.770 |
| Mg (mmol/L) | 0.90±0.07 | 0.89±0.07 | 0.711 |
| GLU (mmol/L) | 5.63±1.32 | 5.36±0.57 | 0.318 |
| UA (umol/L) | 280.18±60.75 | 290.77±67.30 | 0.514 |
| TP (g/L) | 68.90±5.25 | 72.03±4.75 | 0.017* |
| ALB (g/L) | 42.22±4.28 | 43.27±2.89 | 0.265 |
| GLO (g/L) | 26.68±3.68 | 28.77±4.08 | 0.036* |
| CEA (ng/ml) | 2.28±1.34 | 2.34±1.52 | 0.881 |
| CA199 (kU/L) | 9.40±5.25 | 10.43±9.78 | 0.601 |
| AFP (ng/ml) | 2.75±1.40 | 2.50±1.63 | 0.551 |
| CA724 (kU/L) | 2.77±4.34 | 5.04±10.86 | 0.430 |

Supplementary Table 11 Results of blood routine, blood chemistry, and tumor marker between R-Y group and Uncut R-Y without recanalization group at 9^th^ month after surgery. **p*<0.05, ***p*<0.01, ****p*<0.001.

|  | R-Y group  (n=72) | Uncut R-Y without recanalization (n=49) | *p* value |
| --- | --- | --- | --- |
| Hb (g/L) | 130.26±9.99 | 131.68±18.80 | 0.711 |
| RBC (×10^12^/L) | 4.21±0.43 | 4.40±0.49 | 0.066 |
| WBC (×10^9^/L) | 5.59±1.32 | 5.63±1.52 | 0.936 |
| Neu (×10^9^/L) | 3.28±1.22 | 3.28±1.06 | 0.997 |
| Lym (×10^9^/L) | 1.72±0.55 | 1.74±0.51 | 0.894 |
| PLT (×10^9^/L ) | 187.89±39.53 | 185.92±56.03 | 0.845 |
| ALT (U/L) | 25.84±23.47 | 22.41±14.94 | 0.440 |
| AST (U/L) | 26.92±16.03 | 24.38±8.06 | 0.383 |
| TBIL (umol/L) | 11.66±3.80 | 12.63±4.84 | 0.294 |
| DBIL (umol/L) | 4.31±1.76 | 4.45±1.75 | 0.709 |
| IBIL (umol/L) | 7.34±2.37 | 8.17±3.27 | 0.170 |
| BUN (mmol/L) | 5.70±1.08 | 5.78±1.37 | 0.746 |
| CRE (umol/L) | 63.71±12.63 | 65.52±14.25 | 0.535 |
| K (mmol/L) | 4.11±0.55 | 4.32±0.34 | 0.055 |
| Na (mmol/L) | 140.83±1.96 | 141.16±2.39 | 0.499 |
| Cl (mmol/L) | 104.66±1.80 | 104.56±2.25 | 0.832 |
| Ca (mmol/L) | 2.34±0.10 | 2.34±0.12 | 0.873 |
| Mg (mmol/L) | 0.88±0.08 | 0.89±0.10 | 0.447 |
| GLU (mmol/L) | 5.60±1.43 | 5.34±0.52 | 0.311 |
| UA (umol/L) | 295.79±58.28 | 286.59±59.57 | 0.475 |
| TP (g/L) | 71.07±4.90 | 70.44±6.26 | 0.597 |
| ALB (g/L) | 43.04±3.28 | 42.59±4.80 | 0.596 |
| GLO (g/L) | 27.95±4.28 | 27.28±5.60 | 0.521 |
| CEA (ng/ml) | 2.54±1.55 | 2.94±1.56 | 0.234 |
| CA199 (kU/L) | 9.92±4.96 | 11.12±8.17 | 0.387 |
| AFP (ng/ml) | 3.25±2.78 | 2.63±1.72 | 0.244 |
| CA724 (kU/L) | 2.49±2.19 | 2.08±1.94 | 0.478 |

Supplementary Table 12 Results of blood routine, blood chemistry, and tumor marker between R-Y group and Uncut R-Y without recanalization group at 12^th^ month after surgery. **p*<0.05, ***p*<0.01, ****p*<0.001.
